# Molecular Mapping of Water-Stress Responsive Genomic Loci in Lettuce (*Lactuca* spp.) Using Kinetics Chlorophyll Fluorescence, Hyperspectral Imaging and Machine Learning

**DOI:** 10.3389/fgene.2021.634554

**Published:** 2021-02-18

**Authors:** Pawan Kumar, Renee L. Eriksen, Ivan Simko, Beiquan Mou

**Affiliations:** ^1^Crop Improvement and Protection Research Unit, USDA-ARS, Salinas, CA, United States; ^2^Forage Seed and Cereal Research Unit, USDA-ARS, Corvallis, OR, United States

**Keywords:** QTL clusters, water-stress, kinetic chlorophyll fluorescence, hyperspectral imaging, vegetation index, machine learning, random forest, neural network

## Abstract

Deep understanding of genetic architecture of water-stress tolerance is critical for efficient and optimal development of water-stress tolerant cultivars, which is the most economical and environmentally sound approach to maintain lettuce production with limited irrigation. Lettuce (*Lactuca sativa* L.) production in areas with limited precipitation relies heavily on the use of ground water for irrigation. Lettuce plants are highly susceptible to water-stress, which also affects their nutrient uptake efficiency. Water stressed plants show reduced growth, lower biomass, and early bolting and flowering resulting in bitter flavors. Traditional phenotyping methods to evaluate water-stress are labor intensive, time-consuming and prone to errors. High throughput phenotyping platforms using kinetic chlorophyll fluorescence and hyperspectral imaging can effectively attain physiological traits related to photosynthesis and secondary metabolites that can enhance breeding efficiency for water-stress tolerance. Kinetic chlorophyll fluorescence and hyperspectral imaging along with traditional horticultural traits identified genomic loci affected by water-stress. Supervised machine learning models were evaluated for their accuracy to distinguish water-stressed plants and to identify the most important water-stress related parameters in lettuce. Random Forest (RF) had classification accuracy of 89.7% using kinetic chlorophyll fluorescence parameters and Neural Network (NN) had classification accuracy of 89.8% using hyperspectral imaging derived vegetation indices. The top ten chlorophyll fluorescence parameters and vegetation indices selected by sequential forward selection by RF and NN were genetically mapped using a *L. sativa* × *L. serriola* interspecific recombinant inbred line (RIL) population. A total of 25 quantitative trait loci (QTL) segregating for water-stress related horticultural traits, 26 QTL for the chlorophyll fluorescence traits and 34 QTL for spectral vegetation indices (VI) were identified. The percent phenotypic variation (PV) explained by the horticultural QTL ranged from 6.41 to 19.5%, PV explained by chlorophyll fluorescence QTL ranged from 6.93 to 13.26% while the PV explained by the VI QTL ranged from 7.2 to 17.19%. Eight QTL clusters harboring co-localized QTL for horticultural traits, chlorophyll fluorescence parameters and VI were identified on six lettuce chromosomes. Molecular markers linked to the mapped QTL clusters can be targeted for marker-assisted selection to develop water-stress tolerant lettuce.

## Introduction

Drought (water-stress) is a major challenge for crop production around the globe adversely affecting crop growth and productivity. Water stress decreases plants growth by affecting various physiological and biochemical processes such as photosynthesis, chlorophyll synthesis, nutrient metabolism, ion uptake and translocation, respiration, and carbohydrates metabolism (Jaleel et al., 2008; Farooq et al., 2009). Stressed plants display reduced water potential and turgor pressure, stomatal closure and decreased cell enlargement (Farooq et al., 2009) leading to accelerated leaf senescence. Unlike leaf senescence triggered by plant pathogens, water-stress induced leaf senescence is not restrained as local symptoms but involves whole plant (Munné-Bosch and Alegre, 2004). Leaf senescence triggered prematurely under water-stress leads to earlier transition to plant reproductive state (Lim et al., 2007; Behmann et al., 2014) and is characterized by reallocation of nutrients, degradation of leaf pigments and reduction in leaf chlorophyll content. Degradation of leaf chlorophyll leads to diminished photosynthetic efficiency of photosystem II (PSII) and also alters the ratio between reflected, absorbed, and transmitted radiation (Blackburn, 2007).

To ensure global food security, emphasis is placed on enhancing crop productivity which relies largely on breeding crop plants with increased tolerance to water-stress and other abiotic stresses. Genetic mechanisms controlling water-stress tolerance are complex and are often contributed by several traits with polygenic inheritance. In recent years, the rate of genetic improvement has increased due to advances in genotyping technologies. However, exploiting the full potential of advanced molecular tools in water-stress breeding is often limited by our ability to precisely phenotype water-stress related traits. Reduction in photosynthetic efficiency of PS-II and alteration of spectral characteristics can be evaluated by kinetic chlorophyll fluorescence and hyperspectral imaging (Behmann et al., 2014; Yao et al., 2018). Sensor and imaging based non-invasive phenotyping platforms were used in early detection of plant physiological stresses (Petrozza et al., 2014), nutrient deficiency responses (Chaerle et al., 2007), salt stress response (Awlia et al., 2016), and early selection of biotic and abiotic stress-tolerant genotypes (Mir et al., 2012).

Kinetics chlorophyll fluorescence follows quenching kinetics and light curve protocol based on pulse amplitude modulation (PAM) which can probe the performance of the photosynthetic apparatus and evaluate the photosynthetic capacity (Yao et al., 2018). Chlorophyll fluorescence is the light emitted by a green plant tissue when illuminated by light of approximately 400–700 nm, during which blue and red light excite chlorophyll more than green light. The intensity of the emitted light is inversely proportional to the fraction of energy used for photosynthesis, a redox effect (Kalaji et al., 2017). Therefore, the fluorescence signal can be used as a probe for photosynthetic activity of PSII, which is an important component of plant photosynthesis, and it is particularly sensitive to the water stress conditions (Lu and Zhang, 1999). Hyperspectral sensors can identify changes in the spectral signature of the plant under water stress conditions. Under normal conditions plant pigments such as chlorophyll and xanthophyll absorb light in the visible band but reflect most radiance in the near-infrared (NIR) band. Plant under water stress changes the reflectance pattern due to reduced photosynthetic absorbance leading to increased reflectance in the visible spectral band and reduced reflectance in the NIR band range. Several vegetative indices (VI) have been calculated from the ratio of reflectance at different wavelengths that provide additional details for stress detection (Kim et al., 2011). For example, simple ratio or SR (NIR/Red), is often closely related to the leaf area index (LAI) while the normalized differences vegetation index or NDVI (NIR-Red)/(NIR + Red) is often closely related to the green biomass (Nilsson, 1995). Other well-established VIs to detect plant stress are photochemical reflectance index (PRI) (Gamon et al., 1997), red edge inflection point (REIP) (Peñuelas et al., 1993), and carotenoid reflectance index (CRI) (Gitelson et al., 2006).

Water stress leads to a wide range of physiological and biochemical responses in the plant which in turn alters leaf fluorescence and reflectance. Using only one or few fluorescence parameters or VIs may lead to loss of information leading to discriminatory accuracy (Römer et al., 2012), however, on the other hand, including several parameters at a time may be analytically challenging. Machine learning (ML) approaches offer a scalable, modular strategy for data analysis of large sets generated by fluorescence and hyperspectral imaging. ML refers to a group of computerized modeling approaches that can learn patterns from a dataset and discover underlying structures and relationships to explain, predict or classify a new dataset (Singh et al., 2016). ML approaches are particularly important for breeders, physiologists, and pathologists in analyzing large data as it looks at a combination of factors instead of analyzing each trait individually. For example, random forests (RF), neural networks (NN) along with support vector machine (SVM) were applied in identification of biotic and abiotic stress in tomato (Prince et al., 2015), citrus (Garcia-Ruiz et al., 2013), rice (Chen et al., 2014), sugarbeet (Rumpf et al., 2010), chili peppers (Ataş et al., 2012), and oilseed rape (Baranowski et al., 2015).

Lettuce, *Lactuca sativa* L., 2n = 2x = 18, is one of the most important vegetable crops of the United States, is produced under high irrigation conditions. Lettuce consists of up-to 96% water and is greatly dependent on high soil water potential to maintain cell turgidity and palatability (Eriksen et al., 2016). Over 75% of the total lettuce produced in the United States comes from California, which has experienced major drought periods in the past and adequate supply of irrigation water is not guaranteed in the future. Lettuce production could continue to encounter economic losses as water supplies continue to diminish (Eriksen et al., 2016). Genetic basis of water-stress tolerance in lettuce was previously evaluated in a interspecific population derived from *L. sativa* cv. Salinas and its wild progenitor *L. serriola* (Johnson et al., 2000; Uwimana et al., 2012). These two *Lactuca* species are genetically related and are fully cross compatible (Koopman et al., 2001) producing fertile progeny. Using the recombinant inbred line (RIL) progeny derived from this interspecific cross, QTL clusters were identified for various abiotic stresses including water-stress in a study conducted in greenhouse and field experiments (Hartman et al., 2014). Recent availability of the genome sequence of *L. sativa* cv. Salinas (Reyes-Chin-Wo et al., 2017) will facilitate in the identification of candidate genes located in these QTL clusters. Major bottleneck in breeding for water-stress tolerance in lettuce is lack of efficient phenotyping methodologies. Current methodologies are low throughput, destructive, labor-intensive, time-consuming, and error-prone in detecting water-stress at early stages. A semi high-throughput screening method for identifying drought tolerant seedlings in greenhouse was reported which can be used to reduce number of breeding lines with high potential of drought tolerance (Knepper and Mou, 2015). Although, this method is highly effective, it is based on destructive sampling and might not provide insight into plant response to drought progression.

Early detection of stress is critical from plant breeding standpoint as this enables screening of large populations in the given time thereby improving overall efficiency of the whole breeding program. Features from chlorophyll fluorescence, thermography and normalized difference vegetation index were used for early detection of biotic stress in lettuce (Sandmann et al., 2018), however, utilization of these techniques for detection of abiotic stress in lettuce is lacking. The objectives of this study were to evaluate utility of image-based phenotyping for water-stress classification and identify genomic loci of horticultural traits along with the most important chlorophyll fluorescence parameters and vegetation indices selected by machine learning models during water-stress progression and recovery.

## Materials and Methods

### Plant Material and Experimental Layout

An interspecific F_8_ recombinant inbred line (RIL) population (n = 175) derived from a cross between the cultivated lettuce (*L. sativa* cv. Salinas) and its wild relative (*L. serriola* acc. US96UC23) developed at UC Davis, CA (Truco et al., 2013) was used. The cultivated lettuce is a shallow rooted plant which is highly susceptible to water-stress conditions whereas the wild relative, *L. serriola* is a deep-rooted species with greater tolerance to water stress conditions (Gallardo et al., 1996). Two independent water-stress trials were conducted in fall of 2018 and spring of 2019 in a completely randomized design with three replicates under greenhouse conditions. In each trial, ten seeds of each RIL line were germinated for 7 days in plug trays after which six healthy seedlings per line were transplanted into 3.5-inch plastic pots filled with potting soil mix (MiracleGro^®^). Plants were grown in a greenhouse under normal water conditions with temperatures between 15 and 25°C and 12 h of daylight provided with full spectrum lights. Four weeks after transplanting pots were divided into two groups labeled as control (CT) and water stressed (WS). Water-stress was administered by completely withholding water supply to the WS pots while the CT pots were watered every day. Two weeks after the water-stress was initiated, above ground biomass from all plants was harvested and fresh weight (FW) was recorded immediately. All samples were transferred to brown paper bags and oven dried at 60°C for five days to obtain dry weight (DW). Percent water content (WC) was then calculated as [(FW-DW/FW) × 100)]. Descriptive statistics and analysis of variance was conducted using the stats-package of RStudio (RStudio-Team, 2015) and broad-sense heritability (H^2^) for each trait was estimated as V_*g*_/(V_*g*_ + V_*e*_) × 100 where V_*g*_ and V_*e*_ are the genotypic and environmental variances (Wu et al., 2010). In the third experiment conducted in fall of 2019, all RIL lines were grown in a growth chamber (CMP6050, Conviron, Winnipeg, MB, Canada) at 20°C under continuous white light (200 μmol m^–2^ s^–1^) and relative humidity between 50 and 70% throughout the experiment and the water-stress was administered by withholding water supply to plants 2 weeks after transplanting. Plants were phenotyped using chlorophyll fluorescence and hyperspectral camera (see below for details) at four different time intervals (phases): (i) one day before water-stress is initiated (Pre), (ii) 1 week after water-stress (Early), (iii) 2 weeks after water-stress (Late) and (iv) 1 week after re-watering the Late water-stress plants (Recovery).

### Kinetic Chlorophyll Fluorescence Imaging

Chlorophyll fluorescence was measured using a custom-made PAM fluorescence imaging system (Transect FluorCam FC 800; Photon System Instruments, Brno Czech Republic) with progressive scan CCD camera (effective resolution: 1360 × 1024 pixels) and a prime lens (Fujinin HF8XA-1). The light panel (FluorCam SN-FC800-257) consisted of two actinic lights (red-orange (620nm) with max intensity of 415 μmol m^–1^ s^–1^ and a cool white light with max intensities 1,023 μmolm^–1^ s^–1^). The light panel also included pulse-modulated short flashes (red-orange; 620 nm) for accurate measurements of minimal fluorescence (F_0_), a saturating light pulse source (cool white with 4,753 μmol m^–1^ s^–1^) for maximal fluorescence (F_*m*_) detection and a far-red light source (735 nm with max light intensity of 12 μmol m^–1^ s^–1^) for F_0_‘ determination. The distance between the plants and the camera was held constant at 240 mm throughout the experiment. Plants were placed in a dark room for 30 min to open all PSII reaction centers. The kinetic chlorophyll fluorescence (ChlF) curves and images of the dark-adapted plants was acquired following Kautsky effect (Kautsky and Hirsch, 1931; Govindjee, 1995) measured in pulse-amplitude modulated mode (PAM). Briefly, the measurements start in the dark-adapted state by measuring minimal chlorophyll fluorescence (F_0_) by low intensity measuring flashes followed by measuring maximum fluorescence (F_*m*_) by exposing the dark-adapted plants to strong saturating flashes (for 320 ms at 2,300 μ mol m^–1^ s^–1^). Plants are then exposed to a constant actinic light to measure peak-fluorescence (Fp) and a series of saturated flashes are applied to measure instantaneous fluorescence (*Ft_Ln*, *Ft_Lss*) and maximum fluorescence (*Fm_Ln*, *Fm_Lss*) during light adaptation. A dark relaxation period follows the actinic light period during which measurements on instantaneous and maximum fluorescence (*Ft_Dn*, *Fm_Dn*) are recorded. From the basic chlorophyll fluorescence measurements, 50 parameters were calculated [For details on all parameters refer to Supplementary Data 1 (Adhikari et al., 2019)].

### Hyperspectral Imaging

The VNIR hyperspectral imaging unit in the PlantScreen^TM^ (Photon System Instruments, Brno, Czechia) consists of a hyperspectral camera and a uniformly illuminating halogen lamp mounted directly on the chlorophyll fluorescence imaging unit. The VNIR camera had CMOS sensors with 1,920 × 1,000 pixel resolution that can measure reflectance in visible and near infra-red spectrum (350–950 nm). Two sets of calibrations for image acquisition are performed prior to every measurement. The first one is a white calibration where an image is snapped over the calibration plate with lighting. This image is used for homogeneity calibration in additional data processing. The second one is a dark calibration where the image is snapped over the calibration plate but without lighting. This image is used for camera chip dark current subtraction in additional data processing. Plant masks were manually created for each image to extract high quality data from the acquired raw hyperspectral images that were saved as a BIL (band interleaved by lines) format. Vegetative Indices (VI) were calculated from the raw reflectance data using the hsdar package (Lehnert et al., 2018) in RStudio.

### Machine Learning Models

Classification And REgression Training (CARET) (Kuhn, 2015), a R-package for predictive modeling was used for implementing machine learning models in this study. All the phenotypic measurements recorded were partitioned into a training set (80%) and validation set (20%) using *createDataPartition* function and transformed using *preProcess* function. Recursive feature elimination (RFE) algorithm was implemented with these parameters: functions = rfFuncs, method = “repeatedcv,” repeats = 10 for feature selection from the VI, measured, and calculated ChlF parameters. Nine classification models were then built for testing using the features selected with RFE. The models selected for testing were Support Vector Machines with Radial Basis Function Kernel (SVMR) (Method: *svmRadial*; Library: *kernlab*), Random Forest (RF) (Method: *rf*; Library: *randomForest*), Multivariate Adaptive Regression Spline (MARS) (Method: *earth*; Library: *earth*), Neural Network (NN) (Method: *nnet*; Library: *nnet*), k-Nearest Neighbors (KN) (Method: *knn*; Library: *knn*), Self-Organizing Maps (SOM) (Method: *xyf*; Library: *kohonen*), Naive Bayes (NB) (Method: *naive_bayes*; Library: *naivebayes*), Multi-Layer Perceptron (MLP) (Method: *mlp*; Library: *RSNNS*), and Neural Network with Feature Extraction (NN2) (Method: *pcaNNet*; Library: *nnet*). Common control method for building models was the training set, ntrees 2000, resampling method “repeatedcv,” number 10, repeats 10, tuneLength 10. Models were built by inputting all phenotypic variables (ChlF and VI) and the function *varImp* was used to plot the list of top 10 most important variables specific to each model. The models were tested on the validation set (20% of the remainder set) and confusion matrix was generated to estimate accuracy of each model.

### Genetic Linkage Map and QTL Analysis

A high-density genetic linkage map was developed at the University of California, Davis^1^ using lettuce 6.6 million feature Affymetrix high density GeneChip^®^ (Truco et al., 2013). The linkage map consisted of 4,880 SNP markers covering all 9 chromosomes (1,584.86 cM recombinational length) with average density of 3.08 markers per cM. Identification of QTL and estimation of genetic effects were performed by composite interval mapping function implemented in QTL cartographer (Wang et al., 2005). The likelihood ratio (LR) threshold value (α = 0.05) for declaring the presence of QTL was estimated from 1,000 permutations (Doerge and Churchill, 1996). Mapping was performed at 2-cM walk speed in a 10-cM window with five background cofactors, where the cofactors were selected *via* forward–backward stepwise regression method. Quantitative trait loci were defined by one-LOD confidence intervals on either sides of the peak position and were named following a method used in rice (McCouch et al., 1997). Briefly, the QTL is designated as “q” followed by an abbreviation of the trait name, which is then followed by the chromosome name. Multiple QTLs on the same chromosome are distinguished by an alphabetical suffix.

### QTL Cluster and Candidate Genes Analysis

QTL clusters were identified based on co-localization of the horticultural and ChlF or VI QTLs. To identify candidate genes, we integrated the QTL confidence intervals on the genetic map with the lettuce physical map. First the QTL clusters were demarked and flanking SNP markers were identified, then we used the sequences of the flanking SNP markers to search the *L. sativa* genome sequence using BLAST tool to map their physical locations. Functional annotation of the lettuce genome downloaded from the lettuce genome resource^2^ and candidate genes were identified based on the Gene Ontology (GO) enrichment analysis using the Database for Annotation, Visualization and Integrated Discovery (DAVID) v6.8^3^ with Uniprot IDs associated with these genes. GO terms with *P* < 0.05 were considered significantly enriched.

## Results

### Phenotypic Variation in Horticultural Traits

The parents of the RIL mapping population possessed strikingly different phenotypes under well-water and water-stress treatment (Table 1). Under well-watered treatments the average fresh weight and dry weight of Salinas was 63.18 and 3.86 g respectively which was significantly greater than the fresh weight and dry weight of US96UC23 (19.20 and 2.32 g). Salinas also had greater average fresh weight and dry weight (3.22 and 1.59 g) under water-stress conditions compared to US96UC23 (1.20 and 1.05 g). The Salinas parent consisted of significantly higher water content (93.84 and 49.43%) compared to the US96UC23 parent (87.43 and 13.45%) under well-watered and water-stress conditions respectively. All traits displayed normal distribution and transgressive segregation was observed for all traits among RIL lines under well-water and water-stress conditions. Normal distribution of measurements indicates the complex and quantitative nature of these traits under polygenic control. Heritability of horticultural traits ranged from 78.78% for dry weight under water-stress conditions to 81% for fresh weight under well-watered conditions. Statistical parameters of the RIL population grown under well-water and water-stress treatment is presented in Table 1.

**TABLE 1:** Phenotypic values and broad sense heritability (H) estimate of different water-stress related horticultural traits in a lettuce recombinant inbred line (RIL) population (n = 175) of ‘Salinas’ x US96UC23.

Trait	Year	Treatment	Parents			RILs							
			Salinas	UC	Difference	Max	Min	Mean	CV (%)^*a*^	H(%)^*b*^	Norm^*c*^	Skewness	Kurtosis
Fresh weight (g)	2018	Well-Watered	61.76	16.64	45.12***	65.40	18.40	40.12 ± 10.00***	24.93	89.00	0.379	0.09	−0.02
	2019		64.59	21.75	42.84***	70.53	22.23	41.72 ± 9.14***	21.91	91.00	0.460	−0.17	0.52
	2018	Water-Stress	3.60	1.27	2.33***	5.78	1.14	2.79 ± 1.00***	35.84	82.24	2.550	0.92	0.63
	2019		2.84	1.13	1.71***	6.03	1.43	2.95 ± 0.93***	31.53	80.78	0.065	0.70	0.32
Dry weight (g)	2018	Well-Watered	3.27	2.29	0.98**	5.02	1.02	2.68 ± 0.84***	31.34	83.30	0.401	0.19	−0.12
	2019		4.45	2.35	2.1***	5.29	1.13	2.74 ± 0.72***	26.28	80.63	0.112	0.21	0.67
	2018	Water-Stress	1.41	1.13	0.28**	2.22	0.61	1.44 ± 0.35***	24.31	78.78	0.180	0.12	−0.61
	2019		1.76	0.97	0.79***	2.15	0.80	1.47 ± 0.27***	18.37	84.56	0.650	0.11	−0.19
Water content (%)	2018	Well-Watered	94.71	86.88	7.83***	94.73	88.25	93.25 ± 1.66**	1.78	84.30	0.911	0.04	0.13
	2019		92.97	87.98	4.99***	94.12	86.79	92.38 ± 1.28**	1.39	89.30	0.001 nn	−0.71	0.95
	2018	Water-Stress	60.84	11.33	49.51***	92.98	16.49	56.53 ± 19.49**	34.48	78.80	0.007 nn	0.27	−0.77
	2019		38.02	15.56	22.46***	90.68	25.35	54.07 ± 17.06**	31.55	82.89	0.002 nn	0.52	−0.52

** and *** represent significance with *P-*values of 0.01 and 0.001, respectively. ^*a*^Coefficient of Variation. ^*b*^Broad sense heritability. ^*c*^Shapiro-Wilk normality test of residuals. A *p*-value greater than 0.05 indicates normal distribution. Here ‘nn’ represent non-normal distribution of residuals and the data were square root transformed for further analysis.

### Model Selection, Principle Component, and Correlation Analysis

Accuracy to classify validation set was compared between the models by generating model specific confusion matrix for ChlF parameters and VI (Table 2). Random Forest (RF) and Neural Network (NN) outperformed all other models tested with prediction accuracy of RF at 89.66 with 95% confidence interval (CI) of 82.63–94.54 for ChlF parameters and the prediction accuracy of NN at 89.83% (CI: 82.91–94.63) for the VI traits. Top 10 VI and ChlF and VI parameters selected by *varImp* function (Table 3) were used for all downstream analysis in this study.

**TABLE 2:** Machine learning models and their classification accuracies.

Machine Learning Models (library)	Abbr	ChlF				VI			
		Accuracy	Kappa	95% CI		Accuracy	Kappa	95% CI	
				Lower	Upper			Lower	Upper
Random Forest (rf)	RF	0.8966	0.8621	0.8263	0.9454	0.8475	0.7967	0.7697	0.9070
Neural Network (nnet)	NN	0.8362	0.7816	0.7561	0.8984	0.8983	0.8644	0.8291	0.9463
Neural Networks with Feature Extraction (pcaNNet)	NN2	0.8506	0.7759	0.7580	0.9180	0.8750	0.8125	0.7873	0.9359
Support Vector Machines with Radial Basis Function Kernel (svmRadial)	SVMR	0.8362	0.7816	0.7561	0.8984	0.8636	0.7956	0.7739	0.9275
Multi-Layer Perceptron (RSNNS)	MLP	0.7759	0.7011	0.6891	0.8481	0.8644	0.8193	0.7892	0.9205
Multivariate Adaptive Regression Splines (earth)	MARS	0.8448	0.7931	0.7659	0.9054	0.8729	0.8306	0.7990	0.9271
k-Nearest Neighbors (knn)	KN	0.7241	0.6322	0.6334	0.8030	0.8390	0.7855	0.7600	0.9002
Self-Organizing Maps (kohonen)	SOM	0.7328	0.6437	0.6426	0.8107	0.8559	0.8080	0.7794	0.9138
Naive Bayes (nb)	NB	0.6207	0.4943	0.5259	0.7091	0.8051	0.7403	0.7220	0.8722

**TABLE 3:** Description of top ten kinetic chlorophyll fluorescence (ChlF) parameters selected by Random Forest and top ten vegetation indices (VI) selected by Neural Network model.

Type	Abbr	Description
ChlF	NPQ_L4	Non-photochemical quenching induced in light measured at saturating flash 4
ChlF	NPQ_Lss	Steady-state non-photochemical quenching in light
ChlF	QY_D1	Instantaneous PSII quantum yield during dark relaxation measured at far-red flash 1
ChlF	QY_D2	Instantaneous PSII quantum yield during dark relaxation measured at far-red flash 2
ChlF	QY_D3	Instantaneous PSII quantum yield during dark relaxation measured at far-red flash 3
ChlF	QY_L4	Instantaneous PSII quantum yield during light adaptation measured at saturating flash 4
ChlF	QY_Lss	Steady-state PSII quantum yield in light
ChlF	QY_max	Maximum PSII quantum yield. Also known as Fv/Fm
ChlF	Rfd_L4	Instantaneous fluorescence decline ratio in light.
ChlF	Rfd_Lss	Fluorescence decline ratio in steady-state
VI	CRI2	Carotenoid Reflectance Index 2: 1/R515 - 1/R770
VI	Datt5	Datt5: R672/R550
VI	DWSI4	Disease water stress index 4: R550/R680
VI	GDVI_4	Green Difference Vegetation Index 4: (R4800 - R4680)/(R4800 + R4680)
VI	GI	Greenness Index: R554/R677
VI	NDVI	Normalized Difference Vegetation Index: (R800 - R680)/(R800 + R680)
VI	PRI	Photochemical Reflectance Index: (R531 - R570)/(R531 + R570)
VI	PRI_norm	normalized PRI: PRI * (-1)/(RDVI * R700/R670)
VI	SAVI	Soil Adjusted Vegetation Index: (1 + L) * (R800 - R670)/(R800 + R670 + L)
VI	SR	Simple Ratio: R800/R680

**FIGURE 1:**
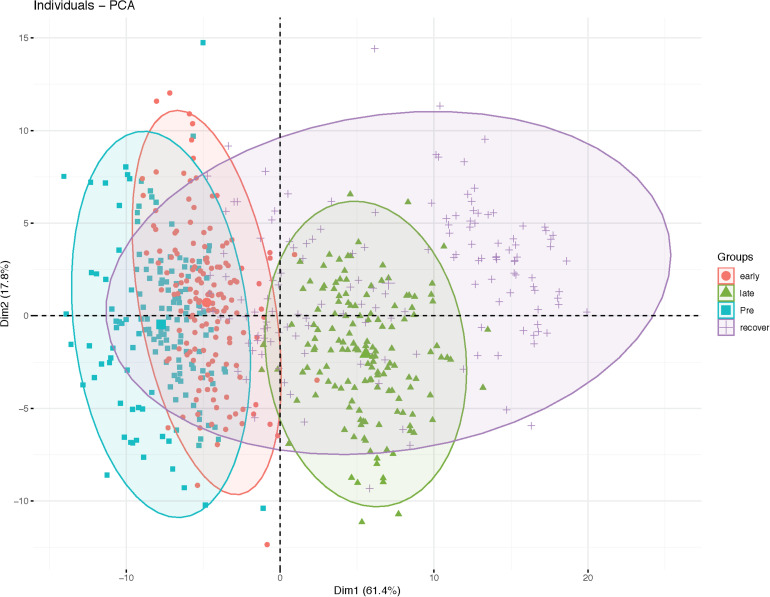
Principal component analysis plot of RIL population during water-stress progression.

Clustering of samples from the training and validation sets were performed by PCA using the selected variables (Figure 1). In the PCA, the first and second dimensions explained 61.4 and 17.8% of the variation respectively. Samples from pre water-stress stress and early water-stress displayed overlapping pattern whereas the samples from late water-stress phase grouped as a distinct cluster. The samples from the recovery phase were more scattered with some clustering with the early or late water-stress phase samples. The first dimension explained the variability in the water-stress progression over time while the second dimension explained variability in the RIL lines during water-stress progression.

**FIGURE 2:**
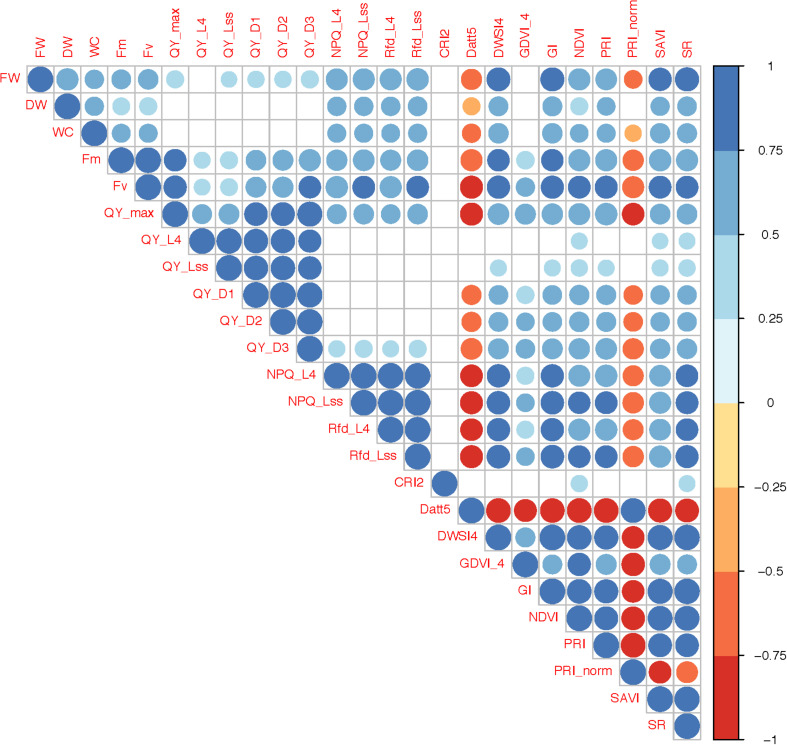
Correlations between horticultural traits, chlorophyll fluorescence parameters and vegetation indices. Only significant correlations (*P*-value <0.05) are presented. The color and size of circles represents direction (positive or negative) and level of significance respectively.

Correlation analysis between horticultural, ChlF and VI traits indicate that the fresh weight (FW) is positively correlated with all other traits except with Datt5 (*r* = −0.68) and PRI_norm (*r* = −0.51) (Figure 2). QY_max, indicator of photosynthetic efficiency of photosystem-II, was positively correlated with most of the traits (*r* = 0.35 to 0.94) and negatively correlated with Datt5 (*r* = −0.80) and PRI_norm (*r* = −0.78), however, it was not correlated with DW, WC, and CRI2. Datt5 and PRI_norm were significantly correlated (*r* = 0.94) and both were negatively correlated with most of the traits. CRI2 was correlated only with NDVI and SR (*r* = 0.45 and 0.43) respectively.

### Parental Variation in ChlF and VI Traits During Water-Stress Progression and Recovery

**FIGURE 3:**
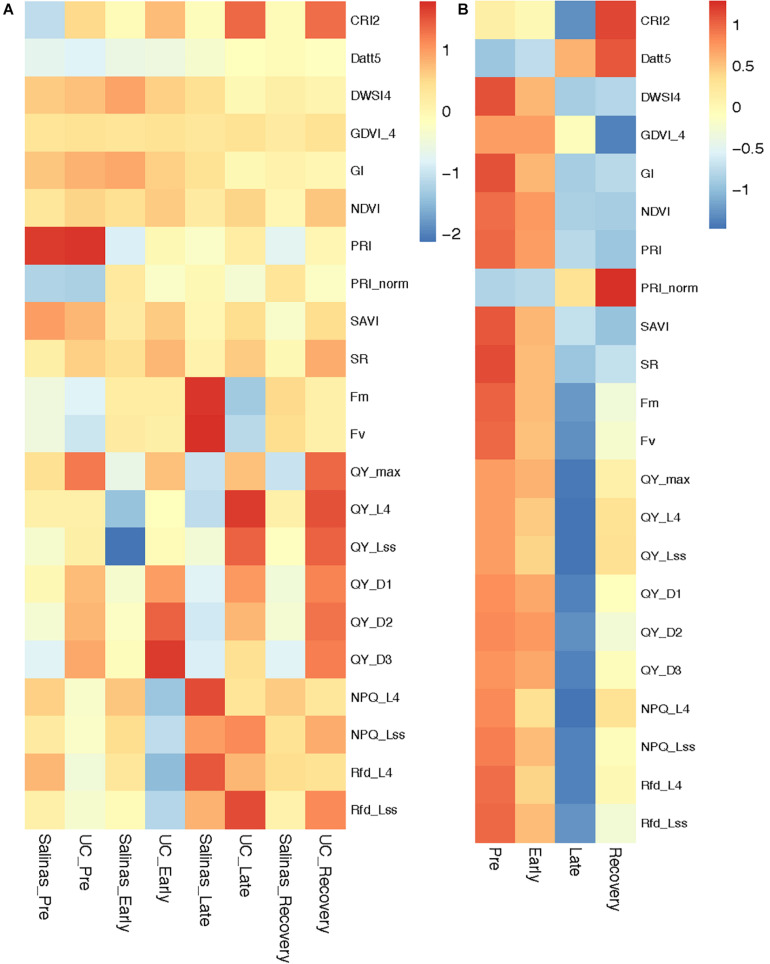
Heatmap depicting changes in chlorophyll fluorescence parameters and vegetation indices during water-stress progression and recovery. **(A)** Mapping parents, **(B)** Recombinant inbred line (RIL) population.

The two mapping parents differed significantly for the selected ChlF and VI traits as water-stress progressed from pre phase to recovery phase. Commonly used ChlF parameters such Fm, Fv along with the ChlF and VI selected by RF and NN models were used to investigate differential responses of Salinas and US96UC23 to water-stress conditions (Figure 3A). No significant differences were observed for Fm and Fv values between the two parents at pre and early phase of the experiment (Supplementary Table 1). At the late phase, Fm and Fv increased by 29 and 31% respectively in ‘Salinas’, whereas Fm and Fv decreased by 11.5 and 4% respectively in US96UC23 indicating that most of the captured energy is dissipated in the form of fluorescence by ‘Salinas’, while US96UC23 is able to use captured energy for photosynthetic processes even under severe water-stress conditions. However, in comparison to the late phase, both Fm and Fv were reduced by an average of 17% in ‘Salinas’ at recovery phase, suggesting partial resumption of photosynthetic processes and efficient utilization of the captured energy (Figure 3A). The mean QY_max values of ‘Salinas’ and US96UC23 under non-stress condition were 0.79 (+0.02) and 0.81 (+0.01) respectively (Supplementary Table 1). In ‘Salinas’, the QY_max was reduced by 3.4% at the early phase which was further decreased by 1.3% (total reduction of 4.7%) at late phase. In US96UC23, QY_max reduced by 1.6% at early stage and no further reduction was observed at the late phase, indicating higher photosynthetic efficiency of PS-II in US96UC23 under water-stress conditions. At recovery phase, QY_max significantly increased by 2.1% in US96UC23 while there was no change in ‘Salinas’. In ‘Salinas’, the non-photochemical quenching of maximum fluorescence (NPQ_L4) steadily increased from 1.35 (±0.33) at pre water-stress phase to 1.37 (±0.02) and 1.54 (±0.15) at early and late phases respectively, whereas in US96UC23 the NPQ values decreased from 1.18 (±0.25) to 0.98 (±0.27) at the early phase then increased to 1.31 (±0.03) at late phase of water-stress. A similar trend was observed for the ratio of fluorescence decline (Rfd) which is an indicator of plant vitality suggesting that ‘Salinas’ has better photo-protective processes and is able to dissipate excess energy under water-stress conditions. There is a significant decline in NPQ and Rfd at recovery phase in both parents indicating that more of captured energy was being used in the photochemical process (photosynthesis) (Supplementary Table 1).

No significant differences were observed for Normalized Difference Vegetation Index (NDVI) at pre and early phase, however, at late phase US96UC23 had NDVI value of 0.80 (±0.01) which was significantly higher (*P* < 0.01) than ‘Salinas’ (0.78 +0.05), similarly Simple Ratio Index (SR) of US96UC23 (9.05 ± 0.49) was significantly higher (*P* < 0.05) than ‘Salinas’ (8.19 ± 0.26). At the recovery phase, NDVI decreased significantly to 0.76 (±0.04) in ‘Salinas’ while it remained constant in US96UC23. The SR values remained constant for ‘Salinas’ at recovery phase, while it was significantly higher (9.39 ± 1.47) in US96CS23, suggesting that US96UC23 remained photosynthetically active for longer durations under water-stress conditions. Variation in the Photochemical Reflectance Index (PRI) was non-significant (*P* < 0.05) between the two parents at pre water-stress phase. A significant difference of 0.013 and 0.008 (*P* < 0.05) was observed between the two parents at early and late phases respectively while a non-significant increase is observed during the recovery phase, suggesting that the changes in carotenoid pigments as a result of water-stress varied significantly between ‘Salinas’ and US96UC23 (Supplementary Table 1).

### Population Variation in ChlF and VI Traits During Water-Stress Progression and Recovery

During the early stages of the water-stress, a relatively smaller reduction was observed in parameters that are indicators of photosynthetic efficiency such as Fm, Fv, QY_max, instantaneous PS-II quantum yield during dark relaxation (QY_Dn), instantaneous PS-II quantum yield during light adaptation (QY_Ln) and coefficients of non-photochemical quenching in light adapted state (qN_Ln) (Figure 3B and Supplementary Table 1). All of these ChlF parameters were further reduced significantly as the plants experienced severe water-stress (late phase). Under severe water stress, non-photochemical quenching in PSII (NPQ) increased due to higher heat dissipation resulting in lower Fm in late phase of water-stress. At the recovery phase many plants experienced severe water-stress as evident by higher non-photochemical quenching in PSII (NPQ) while many plants had overcome the stress and started photosynthesis resulting in increased maximum quantum yield of photosystem-II (QY_max), instantaneous PS-II quantum yield during dark relaxation (QY_Dn), instantaneous PS-II quantum yield during light adaptation (QY_Ln). Water-stress affected reflectance spectra of RIL lines in the visible and near infrared wavelength. The values of VI such as NDVI, SR, and PRI that provide measure of the overall amount and quality of photosynthetic material were decreased as the water stress progressed. The values of other VI such as Plant Senescence Reflectance Index (PSRI), Structure Insensitive Pigment Index (SIPI) and Carotenoid Reflectance Index (CRI), that are indicator of structural and biochemical changes in leaf canopy, were significantly increased as the water-stress progressed. Effect of water-stress progression (pre, early, late and recovery) on ChlF and VI of RIL population is presented as heatmap of the normalized values (Figure 3B).

### Linkage Mapping and QTL Analysis

A total of 25 QTLs were identified for the three horticulturally important traits studied, 15 (60%) were detected in the well-watered (WW) whereas 10 (40%) were detected in water-stress (WS) conditions. Out of the 10 QTLs for fresh weight (FW) that were detected on 6 chromosomes, 6 were detected in WW conditions while 4 were detected in WS conditions (Table 4). Two QTL, *qFW-Chr04* and *qFW-Chr06a*, were consistently detected in both years under WW condition. The phenotypic variation (PV) explained by the fresh weight QTLs in WW conditions ranged from 6.41 to 19.15% whereas PV explained in WS condition ranged from 7.28 to 12.82%. Favorable allele for all FW QTLs were contributed by cultivated lettuce ‘Salinas’. A total of 8 QTLs for dry weight (DW) were detected of which 4 were unique to WW conditions while 3 were identified only under WS conditions. A QTL on chromosome 4 (*qDW-Chr04*) that was identified under both WW and WS conditions but with peak at slightly different position is considered as same QTL due to overlap of one-LOD confidence interval. Five out of the 8 DW QTLs were identified in both 2018 and 2019 indicating that these QTLs are stably expressed across environments. The PV explained by DW QTLs ranged between 7.29 and 16.26% in WW conditions and from 7.32 to 12.61% in WS conditions. Favorable allele for 4 QTLs (*qDW-Chr01b, qDW-Chr04, qDW-Chr07a, qDW-Chr07b*) was contributed by ‘Salinas’ while alleles from the wild parent, US96UC23, increased dry weight at the other 4 QTLs (*qDW-Chr01a, qDW-Chr08, qDW-Chr01c, qDW-Chr03*). A total of 6 QTLs for water content (WC) were identified of which 4 QTLs (*qWC-Chr01a, qWC-Chr01b, qWC-Chr03, qWC-Chr08*) were specific to WW condition while 2 QTLs (*qWC-Chr06a, qWC-Chr06b*) were identified only under WS condition. Five (of 6) WC QTLs had stable expression across environment and were identified both in 2018 and 2019. The PV explained ranged from 6.88 to 12.33% under WW conditions and between 10.76 and 16.70% under WS conditions. Favorable alleles for 3 QTLs (*qWC-Chr01b, qWC-Chr03, qWC-Chr08*) was contributed by ‘Salinas’ while US96UC23 contributed favorable allele for the remaining 3 QTLs. Interestingly, the wild parent improved water content under both WS and WW condition while the cultivated ‘Salinas’ improved water content only under WW conditions (Table 4).

**TABLE 4:** Summary of horticultural QTL identified in two trials of the water-stress experiment.

Trait	Treatment	QTL name	Year^*a*^	Marker Interval (cM)	LOD score	Additive	R^2^ (%)^*b*^	+ ve Allele^*c*^
Fresh weight (g)	Well-Watered	*qFW-Chr03a*	T-1	49.23–55.25	3.79	2.95	7.88	Salinas
	Well-Watered	*qFW-Chr03b*	T-2	63.56–71.37	3.67	2.93	7.68	Salinas
	Well-Watered	*qFW-Chr04*	T-1	98.24–104.74	8.98	5.53	19.15	Salinas
	Well-Watered		T-2	100.45–104.74	5.12	3.21	11.04	Salinas
	Well-Watered	*qFW-Chr06a*	T-1	100.95–111.16	3.66	3.34	7.16	Salinas
	Well-Watered		T-2	103.77–107.05	3.22	2.54	6.86	Salinas
	Well-Watered	*qFW-Chr07a*	T-2	79.87–83.05	3.30	3.17	6.41	Salinas
	Well-Watered	*qFW-Chr08*	T-1	18.01–23.36	3.83	−3.06	8.11	UC
	Water-Stress	*qFW-Chr05*	T-2	225.84–228.87	3.33	0.31	7.28	Salinas
	Water-Stress	*qFW-Chr06b*	T-2	98.28–103.74	4.06	0.29	9.56	Salinas
	Water-Stress	*qFW-Chr06c*	T-1	121.75–125.66	4.07	0.34	12.36	Salinas
	Water-Stress		T-2	124.56–128.22	5.52	0.28	12.82	Salinas
	Water-Stress	*qFW-Chr07b*	T-2	108.06–110.11	3.31	0.28	7.62	Salinas
Dry weight (g)	Well-Watered	*qDW-Chr01a*	T-1	90.49–98.81	4.72	−0.27	9.80	UC
	Well-Watered		T-2	92.55–104.88	3.44	−0.23	7.29	UC
	Well-Watered	*qDW-Chr01b*	T-1	18.57–24.61	3.66	0.32	7.66	Salinas
	Well-Watered		T-2	20.05–24.87	3.56	0.20	7.48	Salinas
	Well-Watered	*qDW-Chr04*	T-1	89.05–100.7	3.89	0.24	7.99	Salinas
	Well-Watered		T-2	92.08–104.74	5.13	0.38	10.75	Salinas
	Well-Watered	*qDW-Chr07a*	T-1	76.68–83.05	4.38	0.35	9.06	Salinas
	Well-Watered	*qDW-Chr08*	T-1	0.01–8.34	7.55	−0.35	16.26	UC
	Well-Watered		T-2	8.25–13.08	3.73	−0.21	7.79	UC
	Water-Stress	*qDW-Chr01c*	T-2	56.56–57.59	3.79	−0.10	8.45	UC
	Water-Stress	*qDW-Chr03*	T-2	115.68–118.04	4.31	−0.21	10.64	UC
	Water-Stress	*qDW-Chr04*	T-1	101.45–106.25	3.20	0.10	8.39	Salinas
	Water-Stress		T-2	102.55–107.01	3.06	0.07	7.32	Salinas
	Water-Stress	*qDW-Chr07b*	T-1	89.63–98.04	5.62	0.12	12.61	Salinas
	Water-Stress		T-2	96.08–100.04	3.55	0.08	9.10	Salinas
Water content (%)	Well-Watered	*qWC-Chr01a*	T-1	19.32–21.79	3.52	−0.49	7.50	UC
	Well-Watered	*qWC-Chr01b*	T-1	51.03–56.02	5.63	0.49	12.28	Salinas
	Well-Watered		T-2	52.73–58.1	3.16	0.45	6.88	Salinas
	Well-Watered	*qWC-Chr03*	T-1	72.87–81.53	4.32	0.40	9.18	Salinas
	Well-Watered		T-2	81.53–90.25	5.67	0.65	12.33	Salinas
	Well-Watered	*qWC-Chr08*	T-1	90.36–95.70	3.82	0.48	8.08	Salinas
	Well-Watered		T-2	94.18–99.15	3.50	0.37	7.59	Salinas
	Water-Stress	*qWC-Chr06a*	T-1	104.77–109.62	4.56	−6.10	10.76	UC
	Water-Stress		T-2	108.07–115.58	7.94	−7.73	17.25	UC
	Water-Stress	*qWC-Chr06b*	T-1	126.08–130.11	7.44	−7.10	16.70	UC
			T-2	125.67–130.11	5.43	−6.71	12.75	UC

*^*a*^T1: Trial conducted in 2018; T2: Trial conducted in 2019. ^*b*^Percentage of total phenotypic variation. ^*c*^Parent contributed favorable allele. Salinas- *L. sativa* cv Salinas; UC- *L. serriola* acc. US96UC23.*

Linkage mapping for the top 10 ChlF traits and top 10 VIs selected by RF and NN models identified 26 ChlF and 34 VI QTLs distributed on 8 lettuce chromosomes. Number of QTLs per trait ranged from 1 QTL (NPQ_L4, QY_L4, PRI_norm) to 8 QTLs (SR). Four ChlF and 6 VI QTLs were identified before the water-stress was administered, 7 ChlF and 13 VI QTLs were identified at early phase of water-stress and linkage mapping at the late water-stress phase identified 8 QTLs each for ChlF and VI traits (Table 5). At recovery phase of the experiment, 8 ChlF and 7 VI QTLs were mapped. The PV explained by the ChlF QTLs ranged from 6.93 to 13.29% whereas it ranged between 7.2 and 17.19% for VI QTLs. Alleles from US96UC23 increased the trait value for 16 (61.5%) ChlF QTLs and ‘Salinas’ allele increased the trait value for 18 (52.9%) VI QTLs.

**TABLE 5:** Summary of QTL affecting kinetic chlorophyll fluorescence parameters and vegetation indices during water-stress progression.

Trait	Type^*a*^	QTL Name	Treatment	Marker Position (cM)	LOD Score	Additive	R2 (%)^*b*^	+ ve Allele^*c*^
NPQ_L4	ChlF	*qNPQ_L4-Chr02*	Recovery	106.89–110.11	3.6353	0.098	8.63	Salinas
NPQ_Lss	ChlF	*qNPQ_Lss-Chr04a*	Recovery	144.23–148.98	3.5476	0.1082	8.48	Salinas
		*qNPQ_Lss-Chr04b*	Late	185.67–196.36	3.7085	–0.1152	9.17	UC
		*qNPQ_Lss-Chr05a*	Early	138.66–141.79	3.6349	–0.1988	8.88	UC
		*qNPQ_Lss-Chr05b*	Early	185.72–192.79	3.0818	0.1131	6.96	Salinas
		*qNPQ_Lss-Chr06*	Late	105.37–113.9	4.1049	–0.1501	10.4	UC
QY_D1	ChlF	*qQY_D1-Chr01a*	Late	39.75–43.81	4.9633	0.1143	12.63	Salinas
		*qQY_D1-Chr01b*	Late	98.97–102.11	3.0432	–0.0678	6.93	UC
		*qQY_D1-Chr04*	Recovery	214.56–216.89	3.6006	–0.0626	9.11	UC
QY_D2	ChlF	*qQY_D2-Chr01*	Late	39.78–42.99	3.3951	0.1062	9.02	Salinas
		*qQY_D2-Chr04a*	Pre	135.63–137.99	3.3088	–0.0066	8.63	UC
		*qQY_D2-Chr04b*	Recovery	214.56–216.89	3.8923	–0.0804	9.78	UC
QY_L4	ChlF	*qQY_L4-Chr04*	Recovery	214.56–216.89	3.8831	–0.0266	9.57	UC
QY_Lss	ChlF	*qQY_Lss-Chr01*	Early	122.36–126.02	3.5701	0.012	8.99	Salinas
		*qQY_Lss-Chr05*	Early	193.75–197.04	3.3239	0.01	8.3	Salinas
QY_max	ChlF	*qQY_max-Chr01*	Recovery	101.25–105.34	3.593	–0.0322	8.81	UC
		*qQY_max-Chr06*	Pre	136.99–139.16	5.2644	0.0062	13.29	Salinas
Rfd_L4	ChlF	*qRfd_L4-Chr04*	Late	185.67–196.36	3.703	–0.1191	9.07	UC
		*qRfd_L4-Chr05a*	Pre	135.99–139.96	3.0642	–0.1379	7.77	UC
		*qRfd_L4-Chr05b*	Early	156.08–160.1	3.107	–0.1296	7.31	UC
		*qRfd_L4-Chr06*	Late	185.72–192.79	3.7949	–0.1503	9.42	UC
Rfd_Lss	ChlF	*qRfd_Lss-Chr05*	Pre	136.84–141.3	3.1708	–0.1333	7.88	UC
		*qRfd_Lss-Chr06a*	Early	30.09–33.96	3.3007	0.1424	7.66	Salinas
		*qRfd_Lss-Chr06b*	Late	185.72–192.79	3.2653	–0.126	8.1	UC
			Recovery	112.39–116.78	3.1871	–0.2023	8.31	UC
		*qRfd_Lss-Chr07*	Early	38.99–41.86	3.1248	0.1407	7.92	Salinas
CRI2	VI	*qCRI2-Chr04*	Recovery	32.49–35.69	3.1143	0.3709	7.9	Salinas
		*qCRI2-Chr05a*	Early	138.25–141.99	6.0043	–0.7174	14.89	UC
		*qCRI2-Chr05b*	Early	179.68–184.11	3.0481	0.3186	7.2	Salinas
		*qCRI2-Chr05c*	Recovery	211.23–215.68	3.1283	0.3772	7.91	Salinas
		*qCRI2-Chr06*	Late	26.01–29.73	3.5644	0.2398	8.82	Salinas
Datt5	VI	*qDatt5-Chr01*	Early	102.5–106.29	4.3535	0.0279	11.43	Salinas
		*qDatt5-Chr03*	Late	52.62–56.01	3.0774	–0.047	7.74	UC
DWSI4	VI	*qDWSI4-Chr03*	Early	42.55–45.01	3.4067	0.1207	8.17	Salinas
		*qDWSI4-Chr05a*	Early	129.86–132.88	6.0003	0.2479	14.86	Salinas
		*qDWSI4-Chr05b*	Early	142.98–146.13	5.7419	–0.2168	14.04	UC
		*qDWSI4-Chr08*	Recovery	98.38–101.81	3.2432	–0.3246	8.5	UC
GDVI_4	VI	*qGDVI_4-Chr03*	Early	118.63–120.99	4.6436	0.0007	17.19	Salinas
		*qGDVI_4-Chr04a*	Late	10.84–14.23	3.72	–0.0058	8.94	UC
		*qGDVI_4-Chr04b*	Late	23.99–26.68	3.1373	0.0052	8.04	Salinas
		*qGDVI_4-Chr05*	Pre	182.43–185.73	4.6902	0.0001	11.81	Salinas
GI	VI	*qGI-Chr05*	Early	142.98–146.13	4.5917	–0.2126	12.04	UC
		*qGI-Chr08*	Recovery	98.38–101.81	3.2193	–0.3358	8.44	UC
NDVI	VI	*qNDVI-Chr01*	Early	139.68–143.74	3.5457	–0.0127	8.89	UC
		*qNDVI-Chr05a*	Recovery	63.53–68.98	3.5837	0.063	9.17	Salinas
		*qNDVI-Chr05b*	Late	106.74–111.45	3.5708	–0.0207	8.34	UC
		*qNDVI-Chr05c*	Pre	182.34–186.98	4.0484	0.0099	10.42	Salinas
PRI	VI	*qPRI-Chr04*	Late	185.62–188.39	4.7341	–0.0085	11.67	UC
		*qPRI-Chr05*	Early	179.68–184.11	4.3706	0.0027	11.18	Salinas
PRI_norm	VI	*qPRI_norm-Chr05*	Early	179.68–184.11	3.1624	–0.0014	7.86	UC
SAVI	VI	*qSAVI-Chr04*	Pre	58.95–63.84	3.152	0.0243	8.54	Salinas
		*qSAVI-Chr05*	Recovery	60.59–64.76	4.3681	0.0601	11.08	Salinas
SR	VI	*qSR-Chr02*	Pre	124.63–128.07	3.1613	0.1168	7.86	Salinas
		*qSR-Chr03a*	Early	28.67–33.45	3.5593	0.4931	8.62	Salinas
		*qSR-Chr03b*	Late	111.05–115.95	3.3169	–0.0858	7.97	UC
		*qSR-Chr05a*	Late	23.78–27.85	4.9192	–0.3354	12.14	UC
		*qSR-Chr05b*	Recovery	64.27–67.93	3.862	0.2548	9.87	Salinas
		*qSR-Chr05c*	Early	115.59–121.64	3.9509	–0.5763	9.63	UC
		*qSR-Chr05d*	Pre	138.25–141.99	3.0818	–0.5367	8.67	UC
		*qSR-Chr05e*	Pre	161.27–166.72	3.4407	0.009	8.2	Salinas

*^*a*^ChlF- Chlorophyll fluorescence; VI- Vegetation indices. ^*b*^Percentage of total phenotypic variation. ^*c*^Parent contributed favorable allele. Salinas- *L. sativa* cv Salinas; UC- *L. serriola* acc. US96UC23.*

### QTL Cluster and Candidate Gene Identification

**FIGURE 4:**
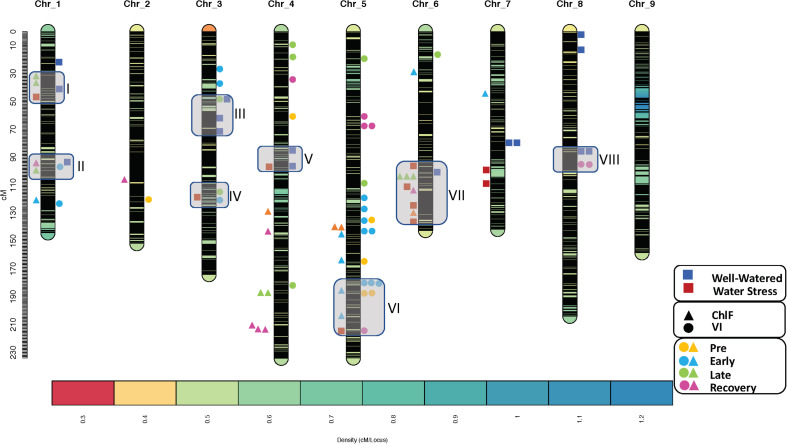
Genomic location of the QTL clusters identified in this study. Color of the legends represent different treatments and the shape of these legends represent phenotypic traits (square: horticultural traits, tringle: chlorophyll fluorescence parameters, and circle: vegetation indices). The boxes on the SNP marker-based lettuce density map represent QTL clusters.

QTL analysis further revealed that QTLs for horticultural traits were mapped along with ChlF or VI QTLs. In total eight co-located QTL clusters were detected, two were identified on the chromosomes 1 and 3 and one each on chromosomes 4, 5, 6, and 8. The size of QTL cluster ranged from 13cM on chromosome 4–51cM on chromosome 6. All clusters consisted of one or more horticultural QTL along with one or more ChlF and/or VI QTLs. The number of QTLs in each cluster ranged from three in cluster IV and V to ten in cluster VII (Figure 4). Most notably, in the cluster II the QTL for maximum quantum yield of photosystem-II (*qQY_max-Chr01*) identified in the recovery phase co-localized with QTL for steady state quantum yield of photosystem-II in dark relaxation (*qQY_D1-Chr01*) and a dry weight QTL, (*qDW-Chr01a*). The additive allele for all these QTLs originated from the wild parent US96UC23 suggesting the importance of this segment of chromosome 1 in improving photosynthetic efficiency of the cultivated lettuce. Co-localization of ChlF and VI QTLs including QTLs for NDVI, GDVI, PRI, NPQ, and QY along with the fresh weight QTL *qFW-Chr05* on chromosome 5 between 180 to 216 cM (cluster VI) signify the role of this segment in affecting chlorophyll content, light harvesting and utilization efficiency, photosynthetic process and overall plant vitality. The QTL cluster VII on chromosome 6 harbored QTL for maximum quantum yield of photosystem-II (*qQY_max-Chr06*) which co-localized with two QTLs for fluorescence decline ratio (*qRfd_L4-Chr06 and qRfd_Lss-Chr06*), a QTL for non-photochemical quenching in PSII (*qNPQ_Lss-Chr06*), along with two horticultural QTLs, *qWC-Chr06b* and *qFW-Chr06c*. Except for *qQY_max-Chr06* all QTLs on this cluster were identified under water-stress conditions and the additive allele originated from US96UC23 (Table 5). Subsequently, we mapped the sequences of the SNP markers flanking each QTL cluster to the *L. sativa* cv Salinas reference genome (version8) at the comparative genomics research platform using CoGe blast search^4^. Topmost enriched biological processes from GO enrichment analysis included protein phosphorylation (GO:0006468), response to abscisic acid (GO:0009737), response to water deprivation (GO:0009414), metabolic process (GO:0008152), photosynthesis (GO:0015979). Candidate genes were selected from the enriched functional annotations involved in important biological function such as photosynthesis, light harvesting (GO:0009765), response to water deprivation, and cell de-toxification (GO:0009636). In total we identified 71 genes on six lettuce chromosomes with the number of candidate genes ranging from 6 genes on chromosome 4–17 genes on chromosome 6. Details of the identified candidate genes along with their physical location, functional annotation and Arabidopsis homologs is presented in the Supplementary Table 2.

## Discussion

Response to water-stress differed considerably between the two mapping parents used in this study. The commercially cultivated lettuce, ‘Salinas’ produced higher fresh weight and dry weight under control and water-stress conditions and had significantly higher water content compared to US96UC23. In contrast, the wild type lettuce produced higher percent biomass under control and water-stressed conditions suggesting efficient photosynthesis and robust carbon assimilation compared to the ‘Salinas’ parent. In a previous study, Gallardo et al. (1996) observed that the *L. serriola* produces greater biomass and has markedly higher photosynthetic rate per unit leaf area and better water use efficiency under control and water-stress conditions than *L. sativa* primarily due to deep taproots that allow the plant to access deeper water sources and maintain higher stomatal conductance. Tolerance to water-stress is a complex trait that involves a dynamic and diverse responses oftentimes controlled by large number of loci each with small genetic effects. In general, plants under normal growth condition absorb more visible light for photosynthesis resulting in lower reflectance value. However, as the plants experience water-stress there is increase in reflectance within the visible range. Visual signs of water-stress effect on plants include leaf curling, shrinking, wilting and decolorization. During early stages of water-stress, plants employ protective strategies such as decrease in photosynthesis, increase in chlorophyll fluorescence and heat emissions (Lichtenthaler, 1996). Under severe water-stress there is increased damage to chlorophyll pigments resulting in drastic changes in leaf absorbance and reflectance.

In this study, supervised learning models were used to identify VI and ChlF parameters that were most informative in classifying genotypic interaction in response to water-stress progression. These simulation models are comprehensive environment for stepwise evaluation of the relative role and importance of each factor separately or in various combinations to identify system-level patterns under different environmental conditions (Grimm and Railsback, 2005; Grimm and Berger, 2016). The parameters picked by random forest and neural network accounted for a large proportion of population variability in response to water-stress progression.

Kinetic chlorophyll fluorescence parameters are important indicators of plant vitality and health of leaf photosynthetic apparatus and can provide accurate diagnostics for detecting and quantifying water-stress tolerance in plants (Percival and Sheriffs, 2002). Strong correlation between ChlF parameters and plant senescence in response to abiotic stress was documented by several researchers (Greaves and Wilson, 1987; Araus and Hogan, 1994; Hakam et al., 2000; Percival and Sheriffs, 2002). Chlorophyll fluorescence imaging can be used as a tool to monitor perturbation in photochemical and non-photochemical processes. Both parents exhibited different ChlF quenching curves suggesting differences in photosynthetic strategies to utilize the absorbed light under water-stress conditions. It appears that the US96UC23 parent is efficient in maintaining homeostasis of photosynthetic machinery under early water-stress stages and thus yielding similar ChlF as at pre water-stress stage. Primary photochemical processes of PSII or the associated QY_max (Fv/Fm) of the cultivated and wild lettuce did not differ at pre and early water-stress stages indicating that it is not an effective indicator of early plant water-stress. On the other hand, traits like Rfd_L3 and Rfd_Lss which indicate potential photosynthetic capacity of the plant differed significantly between the two genotypes at early water-stress stage suggesting that these two traits might be more sensitive than QY_max for detecting early plant water-stress (Lichtenthaler, 1996; Yao et al., 2018). The NPQ_L4 and NPQ_Lss are estimators of photoprotective processes and reflect plant adaptation to counteract stressful environments (Murchie and Lawson, 2013). In the cultivated lettuce, NPQ_L4 and NPQ_Lss increased as the water-stress progressed from pre to late water-stress stage but decreased at the recovery phase indicating that the non-photochemical processes involved in protecting plants under stress were upregulated. However, in wild lettuce, the values of NPQ_L4 and NPQ_Lss dropped at early water-stress but increased rapidly at the late phase suggesting that the photoprotective mechanism in wild lettuce is different from the cultivated lettuce and is upregulated only under severe stress. A similar trend was observed in rose plants subjected to progressive water stress (Gorbe and Calatayud, 2012) where they also found that the non-photochemical processes were upregulated during early water-stress but were downregulated under extreme water stress conditions. The parameter QY_Dn measures the photochemical efficiency of PSII and represents the proportion of light harvested by chlorophyll and utilized in the photochemical reaction in PSII (Yao et al., 2018). A significant reduction in QY_D1 was observed in the cultivated lettuce indicating reduced CO_2_ supply to the chloroplast due to stress-induced stomatal closure (Zhou et al., 2017; Yao et al., 2018) while the QY_D1 was unaffected in the wild lettuce indicating that the photochemical efficiency was maintained under stress condition resulting in higher QY_max in wild lettuce even under water-stress conditions. Eriksen et al. (2020) found higher mesophyll conductance rates in US96UC23, which could allow for increased conductance of CO_2_ supply to the chloroplast during stomatal closure and allow for higher QY_D1.

Plants encountering water stress show higher reflectance values in the visible range than well-watered plants, because the non-stressed plants absorb more light in this range for photosynthesis, therefore having lower reflectance value. The reflectance values in the blue (450–485 nm) and red (525–700 nm) regions were significantly high at late phase of water-stress suggesting reduction in photosynthetic pigment concentration due to water deficit in the leaves. The results indicate that the concentration of primary photosynthetic pigments can be monitored by recording the amount of light reflected in the visible region. The photochemical reflectance index [PRI; Gamon et al. (1997)] and the normalized difference vegetation index [NDVI; Rouse et al. (1974)] are the most commonly used and analyzed indices for crop water stress assessment (Katsoulas et al., 2016). The PRI is sensitive to the epoxidation state of the xanthophyll pigment and photosynthetic efficiency (Gamon et al., 1992, 1997; Ihuoma and Madramootoo, 2017). The changes in xanthophyll are related to the dissipation of the excess energy that cannot be processed through photosynthesis and this interconversion of the xanthophyll cycle pigment can be detected in leaves as subtle changes in reflectance at 531 nm (Gamon et al., 1992, 1997). Sensitivity of PRI for plant water stress detection is well documented in several crops such as tomato (Kittas et al., 2003), spring barley and sugar beet (Borzuchowski and Schulz, 2010), olives (Sun et al., 2008; Marino et al., 2014), soybean and cotton (Inamullah and Isoda, 2005). Significant differences in PRI values between ‘Salinas’ and US96UC23 during water-stress progression suggest diverse mechanisms involved in the energy dissipation under water-stress in the cultivated and wild lettuce. We also found that the PRI is correlated with many other chlorophyll florescence parameters and VI. Similar results were reported by Sarlikioti et al. (2010) where they found a strong correlation between PRI and relative water content, CO_2_ assimilation, stomatal conductance, operating efficiency of PSII and non-photochemical quenching (NPQ) in greenhouse grown tomato plants.

The NDVI is based on the reflectance of leaves in the visible and near-infrared bands of the electromagnetic spectrum and is a numerical indicator of the amount of chlorophyll and vegetation greenness. In this study we found that the NDVI was correlated with horticultural traits including fresh weight and water content and chlorophyll fluorescence parameters such as Fm, Fv and QY_max. High correlation between NDVI and plant biomass, chlorophyll, leaf area and yield in crops plant was reported by several researchers (Jones et al., 2004, 2007; Köksal et al., 2011) suggesting it as a good indicator of nitrogen content and biomass. The NDVI was reported to have a strong correlation with the plant water status in crops like cotton (Yi et al., 2013), grapes (González-Fernández et al., 2015), watermelon (Genc et al., 2011) and apples (Kim et al., 2011). Many other VI analyzed in this study were reported to be correlated to plant growth parameters and plant water content in other crops. For example, simple ratio (SR) and soil adjusted vegetation index (SAVI) were found to be corelated with plant growth in sugar-beet grown under different levels of irrigation (Köksal et al., 2011) and was used for detecting water stress in potatoes (Amatya et al., 2012), and green beans (Köksal, 2011). The green normalized difference vegetation index (GDVI) was found to be a good estimator of water stress for watermelon in semi-arid regions (Genc et al., 2011) whereas PRI_norm (Berni et al., 2009) was found to be significantly correlated with relative water content of tomatoes (Ihuoma and Madramootoo, 2019).

In this study 25 QTL affecting important horticultural traits were identified and evaluated under well-watered and water-stress conditions. We observed that four DW QTL (*qDW-Chr01a*, *qDW-Chr04*, *qDW-Chr07a*, *qDW-Chr08*) were previously reported (Hartman et al., 2014; Kerbiriou et al., 2016) and all the remaining QTL are novel and not previously reported. We found multi-year as well as multi-treatment QTL for the horticultural traits clustered on six of the nine chromosomes. The co-localization of QTLs for different traits is observed in several crops, for example in cabbage 144 QTLs controlling 24 agronomic traits were identified in 12 QTL clusters on eight chromosomes (Lv et al., 2016). A total of 16 QTL-clusters were identified in a wheat double haploid population harboring QTLs controlling chlorophyll content, NDVI and many agronomic traits expressing in response to different water regimes (Shi et al., 2017). The clustering of QTLs for different traits is commonly seen in several crops and might be caused by one or several important genes participating in more than one pathway, for example a single locus Xgwm212 in wheat is associated with biomass production, tillering, and phosphorus absorption and utilization (Zhang et al., 2007). A QTL for DW originating from the ‘Salinas’ parent and identified in recovery treatment was reported on chromosome 05 (Hartman et al., 2014) in a marker interval (65cM to 74cM) where three VI QTL (*qNDVI-Chr05a*, *qSAVI-Chr05*, *qSR-Chr05b*) were mapped in our study. Interestingly, the three VI QTLs were also detected at the recovery phase of the water-stress progression experiment and the alleles from the ‘Salinas’ parent improved the trait. Together, these findings further suggest that proximal non-destructive image-based phenotyping can be effectively applied for water-stress breeding and can provide additional information enabling in better understanding the genetic architecture of water-stress tolerance in lettuce. Recent availability of high-quality lettuce genome sequence gave us opportunity for searching for all potential candidate genes within QTL confidence intervals. Typically, the QTL interval consist of hundreds of genes, many not associated with the trait of interest, therefore selecting candidate genes is based on the overrepresentation in the biological processes. Our search of candidate genes in the QTL interval revealed genes associated with biological processes such as photosynthesis, chlorophyll biosynthesis and survival under water-stress condition. Using similar approach in poplar, candidate genes underlying water-use efficiency were identified in the marker interval harboring QTLs for productivity, architecture and leaf traits (Monclus et al., 2012). Similarly in grapes, Correa et al. (2014) used grapevine reference genome to identify 50 candidate genes from 1,173 genes located in the marker interval of QTLs for rachis architecture. In a noteworthy observation, we found co-localization of NDR1/HIN1-like (NHL25), LEA14, heat shock protein (BIP1), and early dehydration stress responsive gene (ERD4) with chlorophyll and photosynthesis related genes on chromosome 6 where QTLs for fresh weight and water content were detected under water stress conditions. Plant NDR1/HIN1-like (NHL) and late embryo abundant (LEA) genes play crucial role in triggering response to biotic and abiotic stresses in many crops (Bao et al., 2016; Song et al., 2019; Liu et al., 2020). Overexpression of NDR1/HIN1-like gene NHL6 significantly improved drought tolerance in transgenic Arabidopsis (Bao et al., 2016). Heat shock proteins act to protect proteins and membranes and are reported to improve drought tolerance in sorghum (Johnson et al., 2014), poplar (Yer et al., 2016), soybean and tobacco (Valente et al., 2009). The identification of candidate genes in this study is not comprehensive since the genes regulating expression of the candidate genes in the QTL interval may be located elsewhere in the genome and therefore warrants genome-wide expression study. However, the co-localization of horticultural QTL for fresh weight, dry weight, and water content with QTLs for photosynthetic efficiency of photosystem-II (*QY_max*) and greenness (NDVI) along with presence of genes for drought response, photosynthesis and chlorophyll biosynthesis in the marker interval underline the importance the chromosomal segments harboring QTL clusters in improving water-stress tolerance in lettuce.

## Conclusion

We mapped 85 QTL including 25 QTL for horticultural traits, 26 QTL for kinetic chlorophyll fluorescence parameters and 34 QTL for vegetation indices under well-watered and progressive water-stress conditions. We identified candidate genes involved in drought tolerance, photosynthesis and other important biological processes in the marker interval of the 8 QTL clusters. Furthermore, we found that machine learning models are effective in deciphering hidden patterns in vast datasets and can be helpful in identifying relatively important parameters that can be targeted using marker-assisted selection (MAS) in lettuce breeding programs. Major QTL and their clusters identified in this study will be helpful for MAS for water-stress tolerance breeding in lettuce and help in extending our understanding of genetic architecture of water-stress tolerance in lettuce.

## Data Availability Statement

The raw data supporting the conclusions of this article will be made available by the authors, without undue reservation.

## Author Contributions

PK planned and performed experiments, collected and analyzed data, and drafted the manuscript. BM and RLE conceptualized the study and obtained funding for this investigation. IS and BM provided accesses to chlorophyll fluorescence and hyperspectral imaging facility. All authors commented on and approved the final version of the manuscript.

## Conflict of Interest

The authors declare that the research was conducted in the absence of any commercial or financial relationships that could be construed as a potential conflict of interest.

## Footnotes

1 https://michelmorelab.ucdavis.edu

2 https://lgr.genomecenter.ucdavis.edu

3 https://david.ncifcrf.gov/

4 https://genomevolution.org/coge/CoGeBlast.pl?dsgid=37106

## Supplementary Material

Supplementary Table 1 Phenotypic values of selected chlorophyll fluorescence parameters and vegetation indices during drought stress progression.

Supplementary Table 2 Details of the QTL clusters and candidate genes identified in this study.
