# Supplementary material for: Molecular Mapping of Water-Stress Responsive Genomic Loci in Lettuce (Lactuca spp.) Using Kinetics Chlorophyll Fluorescence, Hyperspectral Imaging and Machine Learning
Source: Front Genet. 2021 Feb 18;12:634554. doi: 10.3389/fgene.2021.634554 (PMC7935093; doi:10.3389/fgene.2021.634554)
Supplement: Supplementary Table 1 — Phenotypic values of selected chlorophyll fluorescence parameters and vegetation indices during drought stress progression. [file Table_1.DOCX]

**Supplementary Table 1**. Phenotypic values of selected chlorophyll fluorescence parameters and vegetation indices during drought stress progression.

| **Trait** | **Treatment** | **Parents** | |  | **RILs** | | | | |
| --- | --- | --- | --- | --- | --- | --- | --- | --- | --- |
|  |  | **Salinas** | **UC** |  | **Mean** | **Min** | **Max** | **Kurtosis** | **Skewness** |
| **Fm** | Pre | 5337.22 ± 319.13 | 5014.45 ± 406.82 | ns | 6974.48 ± 964.38 | 3680.18 | 9546.03 | 0.60 | -0.61 |
|  | Early | 6003.52 ± 155.8 | 6037.22 ± 208.21 | ns | 6271.39 ± 936.37 | 5057.89 | 8807.49 | 0.21 | -0.20 |
|  | Late | 7537.08 ± 559.91 | 4499.44 ± 781.43 | *** | 3566.68 ± 1930.51 | 7981.7 | 9050.2 | 0.60 | 1.17 |
|  | Recovery | 6336.5 ± 474.93 | 5956.75 ± 573.22 | *** | 4976.28 ± 2211.12 | 8236.64 | 9256.25 | -1.19 | -0.13 |
| **Fv** | Pre | 4261.33 ± 306.51 | 3847.76 ± 342.47 | * | 5602.5 ± 811.82 | 2965.28 | 7829.67 | 0.42 | -0.50 |
|  | Early | 4909.95 ± 983.2 | 4809.84 ± 159.71 | * | 4940.36 ± 793.39 | 4223.62 | 7164.31 | 0.10 | -0.05 |
|  | Late | 6247.92 ± 416.34 | 3701.49 ± 793.57 | *** | 2225.97 ± 1635.84 | 7136.32 | 7264.04 | 0.73 | 1.12 |
|  | Recovery | 5094.09 ± 676.69 | 4776.21 ± 502.18 | *** | 3836.72 ± 1935.63 | 7008.23 | 7603.29 | -1.27 | -0.12 |
| **QY_max** | Pre | 0.79 ± 0.02 | 0.81 ± 0.01 | * | 0.8 ± 0.02 | 0.73 | 0.83 | 1.65 | -0.84 |
|  | Early | 0.7633 ± 0.02 | 0.7967 ± 0.01 | ** | 0.78 ± 0.02 | 0.13 | 0.82 | 1.77 | -1.00 |
|  | Late | 0.7533 ± 0.01 | 0.7967 ± 0.05 | *** | 0.52 ± 0.18 | 0.67 | 0.79 | -0.57 | -0.58 |
|  | Recovery | 0.7548 ± 0.01 | 0.8133 ± 0.02 | *** | 0.72 ± 0.1 | 0.41 | 0.83 | -0.15 | -0.92 |
| **QY_L4** | Pre | 0.0367 ± 0.03 | 0.0367 ± 0.03 | ns | 0.14 ± 0.04 | 0.05 | 0.25 | -0.37 | 0.38 |
|  | Early | 0.0033 ± 0.05 | 0.03 ± 0.02 | *** | 0.13 ± 0.03 | 0.17 | 0.19 | 0.12 | -0.24 |
|  | Late | 0.01 ± 0.03 | 0.07 ± 0.09 | * | -0.01 ± 0.11 | 0.15 | 0.54 | 0.90 | -1.35 |
|  | Recovery | 0.0367 ± 0.03 | 0.0667 ± 0.02 | * | 0.11 ± 0.08 | 0.25 | 0.46 | 1.75 | -1.27 |
| **QY_Lss** | Pre | 0.0333 ± 0.04 | 0.0533 ± 0.03 | ** | 0.18 ± 0.05 | 0.07 | 0.31 | -0.32 | 0.36 |
|  | Early | -0.0333 ± 0.04 | 0.0433 ± 0.02 | *** | 0.15 ± 0.03 | 0.14 | 0.22 | -0.64 | 0.04 |
|  | Late | 0.03 ± 0.02 | 0.1 ± 0.08 | *** | 0 ± 0.12 | 0.16 | 0.57 | 0.99 | -1.27 |
|  | Recovery | 0.04 ± 0.02 | 0.1 ± 0.03 | *** | 0.15 ± 0.09 | 0.3 | 0.56 | 2.29 | -1.37 |
| **QY_D1** | Pre | 0.6367 ± 0.03 | 0.6733 ± 0.01 | * | 0.72 ± 0.03 | 0.63 | 0.77 | 0.77 | -0.80 |
|  | Early | 0.6233 ± 0.03 | 0.6833 ± 0.02 | * | 0.7 ± 0.04 | 0.21 | 0.76 | 1.16 | -1.02 |
|  | Late | 0.6033 ± 0.01 | 0.6867 ± 0.15 | *** | 0.34 ± 0.25 | 0.71 | 1.05 | -0.48 | -0.55 |
|  | Recovery | 0.6167 ± 0.02 | 0.6933 ± 0.01 | *** | 0.57 ± 0.2 | 0.77 | 0.97 | 2.49 | -1.63 |
| **QY_D2** | Pre | 0.66 ± 0.03 | 0.7033 ± 0.01 | * | 0.74 ± 0.02 | 0.66 | 0.78 | 1.41 | -0.90 |
|  | Early | 0.6667 ± 0.05 | 0.7267 ± 0.01 | *** | 0.73 ± 0.04 | 0.2 | 0.78 | 2.07 | -1.28 |
|  | Late | 0.64 ± 0.03 | 0.7033 ± 0.1 | *** | 0.37 ± 0.26 | 0.73 | 1.07 | -0.35 | -0.62 |
|  | Recovery | 0.66 ± 0.01 | 0.7233 ± 0.01 | *** | 0.55 ± 0.25 | 0.79 | 1.45 | 3.48 | -1.70 |
| **QY_D3** | Pre | 0.6867 ± 0.06 | 0.7367 ± 0.02 | *** | 0.76 ± 0.02 | 0.68 | 0.79 | 3.98 | -1.42 |
|  | Early | 0.7067 ± 0.03 | 0.76 ± 0.01 | *** | 0.74 ± 0.03 | 0.15 | 0.79 | 2.04 | -1.16 |
|  | Late | 0.6833 ± 0.01 | 0.7233 ± 0.07 | *** | 0.43 ± 0.21 | 0.74 | 0.88 | -0.70 | -0.43 |
|  | Recovery | 0.6867 ± 0.01 | 0.7467 ± 0.01 | *** | 0.63 ± 0.14 | 0.62 | 0.8 | 0.21 | -0.99 |
| **NPQ_L4** | Pre | 1.3567 ± 0.33 | 1.1833 ± 0.25 | ** | 1.53 ± 0.35 | 0.76 | 2.32 | -0.78 | -0.04 |
|  | Early | 1.3733 ± 0.2 | 0.9833 ± 0.27 | ** | 1.3 ± 0.37 | 1.96 | 2.35 | -0.04 | 0.05 |
|  | Late | 1.54 ± 0.15 | 1.3133 ± 0.03 | ** | 0.46 ± 0.33 | 1.43 | 1.46 | -0.19 | 0.69 |
|  | Recovery | 1.36 ± 0.01 | 1.3033 ± 0.01 | * | 1.28 ± 0.32 | 1.92 | 2.24 | 0.30 | 0.18 |
| **NPQ_Lss** | Pre | 1.58 ± 0.41 | 1.4833 ± 0.32 | ** | 1.63 ± 0.33 | 0.84 | 2.48 | -0.33 | 0.04 |
|  | Early | 1.63 ± 0.35 | 1.32 ± 0.29 | *** | 1.47 ± 0.39 | 1.85 | 2.34 | -0.36 | -0.21 |
|  | Late | 1.73 ± 0.23 | 1.7633 ± 0.1 | ** | 0.56 ± 0.37 | 1.74 | 1.79 | 0.05 | 0.60 |
|  | Recovery | 1.61 ± 0.25 | 1.7067 ± 0.3 | *** | 1.18 ± 0.36 | 1.9 | 2.26 | -0.47 | 0.22 |
| **Rfd_L4** | Pre | 1.67 ± 0.43 | 1.4033 ± 0.2 | ns | 1.96 ± 0.47 | 0.94 | 2.94 | -0.82 | -0.05 |
|  | Early | 1.57 ± 0.31 | 1.1933 ± 0.33 | *** | 1.65 ± 0.45 | 2.24 | 2.78 | -0.23 | -0.07 |
|  | Late | 1.8167 ± 0.19 | 1.6667 ± 0.1 | * | 0.64 ± 0.38 | 1.76 | 1.86 | 0.12 | 0.86 |
|  | Recovery | 1.6067 ± 0.33 | 1.59 ± 0.07 | * | 1.41 ± 0.6 | 2.78 | 3.13 | -0.73 | 0.42 |
| **Rfd_Lss** | Pre | 1.9233 ± 0.54 | 1.8067 ± 0.37 | ns | 2.22 ± 0.44 | 1.03 | 3.34 | -0.24 | -0.12 |
|  | Early | 1.87 ± 0.41 | 1.6067 ± 0.41 | ** | 1.93 ± 0.49 | 2.41 | 3.11 | -0.32 | -0.35 |
|  | Late | 2.1 ± 0.26 | 2.28 ± 0.07 | ** | 0.77 ± 0.44 | 2.05 | 2.16 | -0.01 | 0.71 |
|  | Recovery | 1.91 ± 0.07 | 2.1767 ± 0.09 | *** | 1.41 ± 0.69 | 2.65 | 2.98 | -1.22 | 0.24 |
| **CRI2** | Pre | 2.971 ± 0.39 | 4.1068 ± 0.27 | *** | 4.657 ± 1.299 | 2.260 | 9.768 | 1.91 | 0.93 |
|  | Early | 3.6862 ± 0.45 | 4.2802 ± 0.54 | *** | 4.487 ± 1.104 | 2.385 | 7.793 | 0.23 | 0.59 |
|  | Late | 3.6598 ± 0.74 | 4.7395 ± 1.36 | *** | 3.064 ± 0.749 | 1.536 | 4.908 | -0.40 | 0.13 |
|  | Recovery | 3.6932 ± 1.16 | 4.7087 ± 1.28 | *** | 5.781 ± 1.3 | 2.544 | 10.013 | 0.18 | 0.09 |
| **Datt5** | Pre | 0.2901 ± 0.02 | 0.2785 ± 0.03 | * | 0.271 ± 0.031 | 0.206 | 0.383 | 0.70 | 0.68 |
|  | Early | 0.2985 ± 0.02 | 0.3047 ± 0.02 | * | 0.338 ± 0.055 | 0.226 | 0.549 | 1.88 | 0.95 |
|  | Late | 0.3225 ± 0.05 | 0.3401 ± 0.13 | ** | 0.77 ± 0.159 | 0.417 | 1.295 | 0.86 | 0.65 |
|  | Recovery | 0.3481 ± 0.03 | 0.3391 ± 0.03 | ns | 0.928 ± 0.482 | 0.261 | 1.700 | -1.68 | 0.09 |
| **DWSI4** | Pre | 3.0838 ± 0.2 | 3.1234 ± 0.24 | ns | 3.399 ± 0.352 | 2.426 | 4.518 | 0.16 | 0.03 |
|  | Early | 3.2439 ± 0.18 | 3.07 ± 0.14 | ** | 2.864 ± 0.412 | 1.785 | 4.030 | 0.44 | 0.19 |
|  | Late | 2.9861 ± 0.24 | 2.776 ± 0.3 | ** | 1.336 ± 0.283 | 0.753 | 2.308 | 1.51 | 0.78 |
|  | Recovery | 2.8601 ± 0.2 | 2.8066 ± 0.2 | ** | 1.441 ± 0.832 | 0.568 | 3.576 | -1.11 | 0.59 |
| **GDVI_4** | Pre | 0.99968 ± 0.000132 | 0.99979 ± 0.00245 | ns | 0.99983 ± 0.000126 | 0.999 | 0.990 | 8.42 | -1.45 |
|  | Early | 0.99965 ± 0.000120 | 0.99978 ± 0.00039 | ** | 0.99956 ± 0.000522 | 0.994 | 0.999 | 2.81 | -1.05 |
|  | Late | 0.99944 ± 0.001112 | 0.99976 ± 0.001132 | ** | 0.983134 ± 0.01307 | 0.918 | 0.998 | 2.17 | -1.02 |
|  | Recovery | 0.99923 ± 0.010601 | 0.99971 ± 0.010122 | ** | 0.955097 ± 0.05696 | 0.751 | 0.999 | 0.84 | -1.30 |
| **GI** | Pre | 3.2689 ± 0.23 | 3.3439 ± 0.29 | ns | 3.595 ± 0.39 | 2.513 | 4.788 | 0.10 | 0.04 |
|  | Early | 3.3761 ± 0.19 | 3.2204 ± 0.15 | ** | 2.987 ± 0.447 | 1.836 | 4.273 | 0.43 | 0.21 |
|  | Late | 3.1079 ± 0.26 | 2.8913 ± 0.32 | ** | 1.369 ± 0.289 | 0.779 | 2.385 | 1.70 | 0.85 |
|  | Recovery | 2.9622 ± 0.29 | 2.918 ± 0.35 | ns | 1.491 ± 0.864 | 0.589 | 3.782 | -1.07 | 0.60 |
| **NDVI** | Pre | 0.7848 ± 0.01 | 0.7995 ± 0 | ns | 0.831 ± 0.025 | 0.749 | 0.874 | 0.44 | -0.66 |
|  | Early | 0.7937 ± 0.03 | 0.8052 ± 0.01 | ns | 0.795 ± 0.035 | 0.630 | 0.870 | 2.75 | -1.03 |
|  | Late | 0.7804 ± 0.05 | 0.8008 ± 0.11 | ** | 0.555 ± 0.068 | 0.376 | 0.736 | -0.11 | 0.06 |
|  | Recovery | 0.7677 ± 0.04 | 0.8068 ± 0.16 | ** | 0.552 ± 0.18 | 0.239 | 0.828 | -1.51 | -0.03 |
| **PRI** | Pre | 0.019 ± 0 | 0.0197 ± 0.01 | ns | 0.011 ± 0.011 | -0.014 | 0.038 | -0.59 | 0.21 |
|  | Early | -0.0212 ± 0.01 | -0.0079 ± 0.01 | ** | 0.002 ± 0.007 | -0.024 | 0.024 | 1.23 | -0.30 |
|  | Late | -0.012 ± 0.01 | -0.0043 ± 0.02 | ** | -0.047 ± 0.017 | -0.090 | -0.006 | 0.17 | 0.29 |
|  | Recovery | -0.0189 ± 0.01 | -0.0071 ± 0.03 | ** | -0.052 ± 0.041 | -0.119 | 0.013 | -1.59 | -0.03 |
| **PRI_norm** | Pre | -0.0071 ± 0 | -0.0073 ± 0 | ns | -0.004 ± 0.004 | -0.014 | 0.006 | -0.49 | -0.20 |
|  | Early | 0.0094 ± 0 | 0.0034 ± 0 | ** | -0.001 ± 0.005 | -0.015 | 0.024 | 2.38 | 1.69 |
|  | Late | 0.0057 ± 0.01 | 0.0021 ± 0.03 | ** | 0.069 ± 0.035 | 0.005 | 0.226 | 2.55 | 0.95 |
|  | Recovery | 0.0103 ± 0.01 | 0.0035 ± 0.02 | *** | 0.135 ± 0.135 | -0.009 | 0.517 | -1.03 | 0.56 |
| **SAVI** | Pre | 0.7723 ± 0 | 0.7612 ± 0 | ns | 0.758 ± 0.063 | 0.589 | 0.911 | -0.25 | 0.28 |
|  | Early | 0.7259 ± 0.02 | 0.7514 ± 0.03 | ** | 0.679 ± 0.047 | 0.526 | 0.762 | 0.91 | -0.82 |
|  | Late | 0.7112 ± 0.05 | 0.7411 ± 0.09 | ** | 0.474 ± 0.053 | 0.324 | 0.625 | 0.21 | 0.19 |
|  | Recovery | 0.6924 ± 0.08 | 0.7425 ± 0.09 | *** | 0.431 ± 0.138 | 0.207 | 0.680 | -1.39 | 0.16 |
| **SR** | Pre | 8.3204 ± 0.58 | 8.9799 ± 0.25 | ns | 11.101 ± 1.704 | 6.967 | 14.838 | -0.51 | 0.06 |
|  | Early | 8.7121 ± 0.05 | 9.2775 ± 0.47 | *** | 9.032 ± 1.649 | 4.412 | 14.407 | 0.48 | 0.31 |
|  | Late | 8.1906 ± 0.26 | 9.0564 ± 0.49 | *** | 3.611 ± 0.766 | 2.204 | 6.580 | 1.99 | 1.09 |
|  | Recovery | 7.9757 ± 1.5 | 9.3834 ± 1.47 | *** | 4.391 ± 2.457 | 1.629 | 10.633 | -0.80 | 0.69 |
