# Supplementary material for: Molecular Mapping of Water-Stress Responsive Genomic Loci in Lettuce (Lactuca spp.) Using Kinetics Chlorophyll Fluorescence, Hyperspectral Imaging and Machine Learning
Source: Front Genet. 2021 Feb 18;12:634554. doi: 10.3389/fgene.2021.634554 (PMC7935093; doi:10.3389/fgene.2021.634554)
Supplement: Supplementary Table 2 — Details of the QTL clusters and candidate genes identified in this study. [file Table_2.DOCX]

Supplementary Table 2. Details of the QTL clusters and candidate genes identified in this study

| **Cluster** | **Chromosome** | **Map location (cM) : Flanking SNP markers** | **Lettuce**  **genes** | **Physical**  **location (bp)** | **Arabidopsis**  **homolog** | **Pfam**  **annotations** | **Panther**  **annotations** | **GO**  **annotations** | **Gene**  **Symbol** | **Gene**  **description** |
| --- | --- | --- | --- | --- | --- | --- | --- | --- | --- | --- |
| **I** | **Chr1** | **28 -56 : AWJR - AYQT** |  |  |  |  |  |  |  |  |
|  |  |  | Ls1_27981.1 | 31621869:31622670 | ATCG00580 | PF00283,PF00284 | PTHR33391,PTHR33391:SF1 | GO:0015979,GO:0046872,GO:0016021,GO:0009523 | psbE | Photosystem II reaction center, Cytochrome b559 subunit alpha |
|  |  |  | Ls1_29260.1 | 33419039:33419799 | AT2G18790 | PF00360 | PTHR24423,PTHR24423:SF563 | GO:0018298,GO:0009584,GO:0006355 | PHYB | Phytochrome B |
|  |  |  | Ls1_32540.1 | 34356484:34356783 | AT4G02770 | PF02531 | PTHR31982,PTHR31982:SF4 | GO:0015979,GO:0009538,GO:0009522 | psaD1 | Photosystem I reaction center subunit II-1, chloroplastic |
|  |  |  | Ls1_34401.1 | 38517489:38519601 | AT3G15840 |  | PTHR32429,PTHR32429:SF9 |  | PIFI | Post-illumination chlorophyll fluorescence increase |
|  |  |  | Ls1_35241.1 | 39972010:39973140 | AT1G52230 | PF03244 | PTHR34787,PTHR34787:SF1 | GO:0015979,GO:0009538,GO:0009522 | PSAH2 | Photosystem I reaction center subunit VI-2, chloroplastic |
|  |  |  | Ls1_35400.1 | 40119853:40121680 | AT4G29080 | PF02309 | PTHR31734,PTHR31734:SF5 | GO:0006355,GO:0005634 | PAP2 | Phytochrome-associated protein 2 |
|  |  |  | Ls1_38720.1 | 44408380:44409276 | ATCG00540 | PF01333,PF16639 | PTHR33288,PTHR33288:SF3 | GO:0031361,GO:0020037,GO:0015979 | petA | Photosynthetic electron transfer A |
|  |  |  | Ls1_38741.1 | 44410041:44410842 | ATCG00580 | PF00283,PF00284 | PTHR33391,PTHR33391:SF1 | GO:0015979,GO:0046872,GO:0016021,GO:0009523 | psbE | Photosystem II reaction center protein E |
|  |  |  | Ls1_41100.1 | 47653560:47658153 | AT2G18790 | PF00360 | PTHR24423,PTHR24423:SF563 | GO:0018298,GO:0009584,GO:0006355 | PHYB | Phytochrome B |
|  |  |  | Ls1_42141.1 | 47993214:47993393 | AT2G29420 | PF02798 | PTHR11260,PTHR11260:SF303 | GO:0005515 | GSTU7 | Glutathione S-transferase tau 7 |
| **II** | **Chr1** | **88-110 : AZDA - BJQY** |  |  |  |  |  |  |  |  |
|  |  |  | Ls1_88080.1 | 116030443:116032873 | ATCG00270 | PF00124 | PTHR33149 | GO:0045156,GO:0019684,GO:0009772 | psbD | Photosystem II reaction center protein D |
|  |  |  | Ls1_88101.1 | 116034958:116037162 | ATCG00340 | PF00223 | PTHR30128,PTHR30128:SF4 | GO:0016021,GO:0015979,GO:0009579,GO:0009522 | psaB | Photosystem I P700 chlorophyll a apoprotein A2 |
|  |  |  | Ls1_88121.1 | 116037188:116039440 | ATCG00350 | PF00223 | PTHR30128,PTHR30128:SF8 | GO:0016021,GO:0015979,GO:0009579,GO:0009522 | psaA | Photosystem I P700 chlorophyll a apoprotein A1 |
|  |  |  | Ls1_95341.1 | 126034918:126036012 | AT1G29930 | PF00504 | PTHR21649,PTHR21649:SF26 | GO:0016020,GO:0009765 | LHCB1.3 | Light-harvesting complex protein 1 |
| **III** | **Chr3** | **47-70 : ALHP - AOSA** |  |  |  |  |  |  |  |  |
|  |  |  | Ls3_39480.1 | 52764631:52765413 | AT1G03600 | PF13326 | PTHR34041,PTHR34041:SF1 | GO:0010207 | PSB27-1 | Photosystem II repair protein PSB27-H1, chloroplastic |
|  |  |  | Ls3_41481.1 | 53029402:53031462 | AT1G03630 | PF00106 | PTHR24322,PTHR24322:SF393 | GO:0016491,GO:0008152 | PORC | Protochlorophyllide reductase C, chloroplastic |
|  |  |  | Ls3_45960.1 | 58622238:58623764 | ATCG00680 | PF00421 | PTHR33180,PTHR33180:SF8 | GO:0019684,GO:0016168,GO:0016020 | psbB | Photosystem II CP47 reaction center protein |
|  |  |  | Ls3_50240.1 | 64312894:64315940 | AT1G10360 | PF13417 | PTHR11260,PTHR11260:SF312 | GO:0005515 | GSTU18 | Glutathione S-transferase U18 |
|  |  |  | Ls3_50200.1 | 64361158:64363706 | AT1G10370 | PF13410,PF13417 | PTHR11260,PTHR11260:SF312 | GO:0005515 | GSTU17 | Glutathione S-transferase U17 |
|  |  |  | Ls3_53040.1 | 71027128:71027505 | ATCG00270 | PF00124 | PTHR33149,PTHR33149:SF1 | GO:0045156,GO:0019684,GO:0009772 | psbD | Photosystem II D2 protein |
|  |  |  | Ls3_61721.1 | 73100435:73103400 | AT2G46970 | PF00010 | PTHR12565 | GO:0046983 | PIL1 | Phytochrome interacting factor 3-like 1 |
|  |  |  | Ls3_60001.1 | 77348462:77351244 | AT1G25540 | PF11265 | PTHR12433,PTHR12433:SF11 | GO:0009909,GO:0010114 | PFT1 | Phytochrome and flowering time regulatory protein |
| **IV** | **Chr3** | **109-125 : BGFO - AGBV** |  |  |  |  |  |  |  |  |
|  |  |  | Ls3_101461.1 | 160995871:160997661 | ATCG00270 | PF00124 | PTHR33149 | GO:0045156,GO:0019684,GO:0009772 | psbD | Photosystem II reaction center protein D |
|  |  |  | Ls3_104461.1 | 171805670:171807696 | AT2G17840 | PF06911 | PTHR21068,PTHR21068:SF22 | GO:0009414 | ERD7 | Early-responsive to dehydration 7, chloroplastic |
|  |  |  | Ls3_104200.1 | 174509697:174509990 | ATCG00020 | PF00124 | PTHR33149,PTHR33149:SF6 | GO:0045156,GO:0019684,GO:0009772 | psbA | Photosystem II reaction center protein A |
| **V** | **Chr4** | **79-92 AFKB - BMWP** |  |  |  |  |  |  |  |  |
|  |  |  | Ls4_51841.1 | 76708803:76709342 | ATCG00020 | PF00124 | PTHR33149,PTHR33149:SF6 | GO:0045156,GO:0019684,GO:0009772 | psbA | Photosystem II protein D1 |
|  |  |  | Ls4_58161.1 | 87243372:87243956 | ATCG00270 | PF00124 | PTHR33149,PTHR33149:SF1 | GO:0045156,GO:0019684,GO:0009772 | psbD | Photosystem II reaction center protein D |
|  |  |  | Ls4_61561.1 | 93650840:93656090 | AT4G22120 | PF14703,PF13967 | PTHR13018,PTHR13018:SF46 | GO:0016020 | CSC1 | Calcium permeable stress-gated cation channel 1 |
|  |  |  | Ls4_72580.1 | 114717746:114719920 | AT5G02500 | PF00012 | PTHR19375,PTHR19375:SF194 |  | HSP70-1 | Heat shock 70 kDa protein 1 |
|  |  |  | Ls4_72600.1 | 114729310:114731461 | AT5G02500 | PF00012 | PTHR19375,PTHR19375:SF194 |  | HSP70-1 | Heat shock 70 kDa protein 1 |
|  |  |  | Ls4_77360.1 | 122575121:122578020 | AT1G56280 | PF05605 | PTHR31875,PTHR31875:SF3 | GO:0009414 | DI19-1 | Drought-induced 19 |
| **VI** | **Chr5** | **188-216 : AZBT - BYVL** |  |  |  |  |  |  |  |  |
|  |  |  | Ls5_141361.1 | 270887691:270888680 | AT2G31670 | PF07876 | PTHR33178,PTHR33178:SF3 |  | UP3 | Stress responsive alpha-beta barrel domain protein |
|  |  |  | Ls5_141381.1 | 270892548:270893126 | AT2G31670 | PF07876 | PTHR33178,PTHR33178:SF3 |  | UP3 | Stress responsive alpha-beta barrel domain protein |
|  |  |  | Ls5_142940.1 | 273066703:273068461 | AT2G26150 | PF00447 | PTHR10015,PTHR10015:SF139 | GO:0043565,GO:0006355,GO:0005634 | HSFA2 | heat shock transcription factor A2 |
|  |  |  | Ls5_146120.1 | 275331111:275332011 | AT5G06760 | PF03760 | PTHR33493,PTHR33493:SF2 | GO:0009790 | LEA46 | Late Embryogenesis Abundant protein invoved in dehydration tolerance |
|  |  |  | Ls5_145201.1 | 276373003:276373649 | AT5G56550 |  | PTHR33172,PTHR33172:SF2 | GO:0006979 | OXS3 | oxidative stress 3 |
|  |  |  | Ls5_145081.1 | 276597819:276600613 | AT3G54510 | PF13967,PF02714 | PTHR13018,PTHR13018:SF30 | GO:0016020 | ERD4 | Early-responsive to dehydration stress protein |
|  |  |  | Ls5_144981.1 | 276683070:276688043 | AT1G29930 | PF00504 | PTHR21649,PTHR21649:SF26 | GO:0016020,GO:0009765 | CAB1 | Chlorophyll A/B binding protein 1 |
|  |  |  | Ls5_144940.1 | 276729474:276739087 | AT1G29930 | PF00504 | PTHR21649,PTHR21649:SF26 | GO:0016020,GO:0009765 | CAB140 | Chlorophyll A/B binding protein 1 |
|  |  |  | Ls5_150761.1 | 283661353:283662299 | AT3G51810 | PF00477 | PTHR34671,PTHR34671:SF1 | GO:0009737 | EM1 | Stress induced protein |
|  |  |  | Ls5_150741.1 | 283709508:283713013 | AT1G21270 | PF07645,PF13947 | PTHR27005,PTHR27005:SF32 | GO:0005509,GO:0030247,GO:0006468 | WAK2 | wall-associated kinase rpotein involved in cellular water homeostasis |
|  |  |  | Ls5_152640.1 | 287620872:287622542 | ATCG00340 | PF00223 | PTHR30128,PTHR30128:SF4 | GO:0016021,GO:0015979,GO:0009579,GO:0009522 | PsaB | Photosystem I P700 chlorophyll a apoprotein A2 |
|  |  |  | Ls5_152620.1 | 287622568:287623593 | ATCG00350 | PF00223 | PTHR30128,PTHR30128:SF8 | GO:0016021,GO:0015979,GO:0009579,GO:0009522 | PsaA | Photosystem I P700 chlorophyll a apoprotein A1 |
|  |  |  | Ls5_153061.1 | 288936415:288937670 | AT3G08940 | PF00504 | PTHR21649,PTHR21649:SF6 | GO:0016020,GO:0009765 | LHCB4.2 | Light harvesting complex photosystem II |
|  |  |  | Ls5_160961.1 | 298974975:298976706 | AT2G41430 | PF07145 | PTHR33790,PTHR33790:SF1 | GO:0009414 | ERD15 | Dehydration-induced protein |
|  |  |  | Ls5_170261.1 | 313072111:313072802 | ATCG00720 | PF00033 | PTHR19271,PTHR19271:SF8 | GO:0022904,GO:0016020 | petB | Photosynthetic electron transfer B |
|  |  |  | Ls5_176141.1 | 321199226:321199477 | ATCG00580 | PF00283,PF00284 | PTHR33391,PTHR33391:SF1 | GO:0015979,GO:0046872,GO:0016021,GO:0009523 | psbE | Cytochrome b559 subunit alpha |
| **VII** | **Chr6** | **89-140 : BCRJ - BGSO** |  |  |  |  |  |  |  |  |
|  |  |  | Ls6_72700.1 | 119610613:119611430 | AT3G20600 | PF03096 | PTHR31852:SF0 | GO:0009626 | NDR1 | Prorein NDR1 involved in biotic and abiotic stress resistance |
|  |  |  | Ls6_72520.1 | 119878482:119879433 | AT5G36970 | PF03096 | PTHR31415 | GO:0009863 | NHL25 | NDR1/HIN1-like 25 |
|  |  |  | Ls6_71841.1 | 120826211:120827200 | AT3G20600 | PF03096 | PTHR31852:SF0 | GO:0009626 | NDR1 | Prorein NDR1 involved in biotic and abiotic stress resistance |
|  |  |  | Ls6_73800.1 | 121422363:121429624 | AT1G32090 | PF14703,PF13967 | PTHR13018,PTHR13018:SF47 | GO:0016020 | ERD4 | Early-responsive to dehydration stress protein |
|  |  |  | Ls6_76240.1 | 128293253:128293777 | AT3G22840 | PF00504 | PTHR14154,PTHR14154:SF5 | GO:0010380,GO:0071486 | ELIP1 | Early light-induced protein 1, chloroplastic, Chlorophyll A-B binding family protein |
|  |  |  | Ls6_76700.1 | 128386052:128386797 | AT3G22840 | PF00504 | PTHR14154,PTHR14154:SF5 | GO:0010380,GO:0071486 | ELIP1 | Early light-induced protein 1, chloroplastic, Chlorophyll A-B binding family protein |
|  |  |  | Ls6_76760.1 | 128426050:128426574 | AT3G22840 | PF00504 | PTHR14154,PTHR14154:SF5 | GO:0010380,GO:0071486 | ELIP1 | Early light-induced protein 1, chloroplastic, Chlorophyll A-B binding family protein |
|  |  |  | Ls6_76441.1 | 128545404:128546062 | AT3G22840 | PF00504 | PTHR14154,PTHR14154:SF5 | GO:0010380,GO:0071486 | ELIP1 | Early light-induced protein 1, chloroplastic, Chlorophyll A-B binding family protein |
|  |  |  | Ls6_75760.1 | 128647813:128648661 | AT3G22840 | PF00504 | PTHR14154,PTHR14154:SF5 | GO:0010380,GO:0071486 | ELIP1 | Early light-induced protein 1, chloroplastic, Chlorophyll A-B binding family protein |
|  |  |  | Ls6_75800.1 | 128672554:128677562 | AT3G22840 | PF00504 | PTHR14154,PTHR14154:SF5 | GO:0010380,GO:0071486 | ELIP1 | Early light-induced protein 1, chloroplastic, Chlorophyll A-B binding family protein |
|  |  |  | Ls6_75900.1 | 128740984:128741574 | AT3G22840 | PF00504 | PTHR14154,PTHR14154:SF5 | GO:0010380,GO:0071486 | ELIP1 | Early light-induced protein 1, chloroplastic, Chlorophyll A-B binding family protein |
|  |  |  | Ls6_79200.1 | 130092813:130093013 | AT4G02770 | PF02531 | PTHR31982,PTHR31982:SF4 | GO:0015979,GO:0009538,GO:0009522 | psaD1 | Photosystem I reaction center subunit II-1, chloroplastic |
|  |  |  | Ls6_83581.1 | 137948566:137951853 | AT5G28540 | PF00012 | PTHR19375,PTHR19375:SF228 |  | BIP1 | heat shock protein 70 (Hsp 70) family protein |
|  |  |  | Ls6_83520.1 | 137995823:137998823 | AT5G42020 | PF00012 | PTHR19375,PTHR19375:SF228 |  | BIP1 | Heat shock protein 70 (Hsp 70) family protein |
|  |  |  | Ls6_84881.1 | 142244166:142244923 | AT1G64065 | PF03168 | PTHR31852,PTHR31852:SF10 |  | LEA14 | Late embryogenesis abundant hydroxyproline-rich glycoprotein. Probable desiccation related |
|  |  |  | Ls6_96860.1 | 158483294:158484771 | AT1G77090 | PF01789 | PTHR31407,PTHR31407:SF18 | GO:0019898,GO:0015979 | PPD4 | Photosystem II reaction center PsbP family protein |
|  |  |  | Ls6_115061.1 | 187967661:187969866 | AT3G05700 | PF14571,PF05605 | PTHR31875,PTHR31875:SF6 | GO:0009414,GO:0009737 | DI9-3 | Drought-responsive dehydration induced 19 homolog 3 |
| **VIII** | **Chr8** | **85-102 : BHCB - AYKE** |  |  |  |  |  |  |  |  |
|  |  |  | Ls8_77880.1 | 111399564:111400410 | ATCG00680 | PF00421 | PTHR33180,PTHR33180:SF8 | GO:0019684,GO:0016168 | psbB | Photosystem II CP47 reaction center protein |
|  |  |  | Ls8_80661.1 | 117958899:117960900 | AT3G54050 | PF00316 | PTHR11556,PTHR11556:SF16 | GO:0042578,GO:0042132,GO:0005975 | HCEF1 | High cyclic electron flow 1 |
|  |  |  | Ls8_83020.1 | 119199299:119201637 | AT2G41430 | PF07145 | PTHR33790,PTHR33790:SF1 | GO:0009414,GO:0009737 | ERD15 | Dehydration-induced protein |
|  |  |  | Ls8_85741.1 | 124402780:124404251 | AT1G19150 | PF00504 | PTHR21649,PTHR21649:SF5 | GO:0016020,GO:0009765 | LHCA6 | Photosystem I chlorophyll a/b-binding protein 6, chloroplastic |
|  |  |  | Ls8_86961.1 | 127775432:127776548 | AT5G56550 |  | PTHR33172,PTHR33172:SF2 | GO:0006979 | OXS3 | oxidative stress 3 |
|  |  |  | Ls8_90440.1 | 130532077:130532995 | AT2G20260 | PF02427 | PTHR34549,PTHR34549:SF2 | GO:0015979,GO:0009538,GO:0009522 | PSAE2 | Photosystem I reaction center subunit IV B, chloroplastic |
|  |  |  | Ls8_91180.1 | 134564989:134565423 | ATCG00350 | PF00223 | PTHR30128,PTHR30128:SF8 | GO:0016021,GO:0015979,GO:0009579,GO:0009522 | PsaA | Photosystem I P700 chlorophyll a apoprotein A1 |
